# A genome for *Cissus* illustrates features underlying its evolutionary success in dry savannas

**DOI:** 10.1093/hr/uhac208

**Published:** 2022-09-13

**Authors:** Haiping Xin, Yi Wang, Qingyun Li, Tao Wan, Yujun Hou, Yuanshuang Liu, Duncan Kiragu Gichuki, Huimin Zhou, Zhenfei Zhu, Chen Xu, Yadong Zhou, Zhiming Liu, Rongjun Li, Bing Liu, Limin Lu, Hongsheng Jiang, Jisen Zhang, Junnan Wan, Rishi Aryal, Guangwan Hu, Zhiduan Chen, Robert Wahiti Gituru, Zhenchang Liang, Jun Wen, Qingfeng Wang

**Affiliations:** Core Botanical Gardens/Wuhan Botanical Garden, Chinese Academy of Sciences, Wuhan 430074, China; CAS Key Laboratory of Plant Germplasm Enhancement and Specialty Agriculture, Wuhan Botanical Garden, Chinese Academy of Sciences, Wuhan 430074, China; Sino-Africa Joint Research Center, Chinese Academy of Sciences, Wuhan 430074, China; CAS Key Laboratory of Plant Resources, Institute of Botany, Chinese Academy of Science, Beijing 100093, China; Core Botanical Gardens/Wuhan Botanical Garden, Chinese Academy of Sciences, Wuhan 430074, China; CAS Key Laboratory of Plant Germplasm Enhancement and Specialty Agriculture, Wuhan Botanical Garden, Chinese Academy of Sciences, Wuhan 430074, China; Sino-Africa Joint Research Center, Chinese Academy of Sciences, Wuhan 430074, China; University of Chinese Academy of Sciences, Beijing 100049, China; Core Botanical Gardens/Wuhan Botanical Garden, Chinese Academy of Sciences, Wuhan 430074, China; Sino-Africa Joint Research Center, Chinese Academy of Sciences, Wuhan 430074, China; Key Laboratory of Southern Subtropical Plant Diversity, Fairy Lake Botanical Garden, Shenzhen & Chinese Academy of Science, Shenzhen 518004, China; Core Botanical Gardens/Wuhan Botanical Garden, Chinese Academy of Sciences, Wuhan 430074, China; University of Chinese Academy of Sciences, Beijing 100049, China; Core Botanical Gardens/Wuhan Botanical Garden, Chinese Academy of Sciences, Wuhan 430074, China; University of Chinese Academy of Sciences, Beijing 100049, China; Core Botanical Gardens/Wuhan Botanical Garden, Chinese Academy of Sciences, Wuhan 430074, China; University of Chinese Academy of Sciences, Beijing 100049, China; Core Botanical Gardens/Wuhan Botanical Garden, Chinese Academy of Sciences, Wuhan 430074, China; University of Chinese Academy of Sciences, Beijing 100049, China; Core Botanical Gardens/Wuhan Botanical Garden, Chinese Academy of Sciences, Wuhan 430074, China; University of Chinese Academy of Sciences, Beijing 100049, China; Core Botanical Gardens/Wuhan Botanical Garden, Chinese Academy of Sciences, Wuhan 430074, China; Core Botanical Gardens/Wuhan Botanical Garden, Chinese Academy of Sciences, Wuhan 430074, China; CAS Key Laboratory of Plant Germplasm Enhancement and Specialty Agriculture, Wuhan Botanical Garden, Chinese Academy of Sciences, Wuhan 430074, China; Sino-Africa Joint Research Center, Chinese Academy of Sciences, Wuhan 430074, China; Key Laboratory of Southern Subtropical Plant Diversity, Fairy Lake Botanical Garden, Shenzhen & Chinese Academy of Science, Shenzhen 518004, China; Core Botanical Gardens/Wuhan Botanical Garden, Chinese Academy of Sciences, Wuhan 430074, China; CAS Key Laboratory of Plant Germplasm Enhancement and Specialty Agriculture, Wuhan Botanical Garden, Chinese Academy of Sciences, Wuhan 430074, China; Sino-Africa Joint Research Center, Chinese Academy of Sciences, Wuhan 430074, China; Sino-Africa Joint Research Center, Chinese Academy of Sciences, Wuhan 430074, China; State Key Laboratory of Systematic and Evolutionary Botany, Institute of Botany, Chinese Academy of Science, Beijing 100093, China; Sino-Africa Joint Research Center, Chinese Academy of Sciences, Wuhan 430074, China; State Key Laboratory of Systematic and Evolutionary Botany, Institute of Botany, Chinese Academy of Science, Beijing 100093, China; Core Botanical Gardens/Wuhan Botanical Garden, Chinese Academy of Sciences, Wuhan 430074, China; Center for Genomics and Biotechnology, Haixia Institute of Science and Technology, Fujian Agriculture and Forestry University, Fuzhou 350002, China; Core Botanical Gardens/Wuhan Botanical Garden, Chinese Academy of Sciences, Wuhan 430074, China; CAS Key Laboratory of Plant Germplasm Enhancement and Specialty Agriculture, Wuhan Botanical Garden, Chinese Academy of Sciences, Wuhan 430074, China; Sino-Africa Joint Research Center, Chinese Academy of Sciences, Wuhan 430074, China; Department of Horticultural Science, North Carolina State University, Raleigh, NC 27695, USA; Core Botanical Gardens/Wuhan Botanical Garden, Chinese Academy of Sciences, Wuhan 430074, China; Sino-Africa Joint Research Center, Chinese Academy of Sciences, Wuhan 430074, China; Sino-Africa Joint Research Center, Chinese Academy of Sciences, Wuhan 430074, China; State Key Laboratory of Systematic and Evolutionary Botany, Institute of Botany, Chinese Academy of Science, Beijing 100093, China; Department of Botany, Jomo Kenyatta University of Agriculture and Technology, 62000-00200, Nairobi, Kenya; Sino-Africa Joint Research Center, Chinese Academy of Sciences, Wuhan 430074, China; CAS Key Laboratory of Plant Resources, Institute of Botany, Chinese Academy of Science, Beijing 100093, China; Department of Botany, National Museum of Natural History, Smithsonian Institution, Washington DC 20013-7012, USA; Core Botanical Gardens/Wuhan Botanical Garden, Chinese Academy of Sciences, Wuhan 430074, China; CAS Key Laboratory of Plant Germplasm Enhancement and Specialty Agriculture, Wuhan Botanical Garden, Chinese Academy of Sciences, Wuhan 430074, China; Sino-Africa Joint Research Center, Chinese Academy of Sciences, Wuhan 430074, China

## Abstract

*Cissus* is the largest genus in Vitaceae and is mainly distributed in the tropics and subtropics. Crassulacean acid metabolism (CAM), a photosynthetic adaptation to the occurrence of succulent leaves or stems, indicates that convergent evolution occurred in response to drought stress during species radiation. Here we provide the chromosomal level assembly of *Cissus rotundifolia* (an endemic species in Eastern Africa) and a genome-wide comparison with grape to understand genome divergence within an ancient eudicot family. Extensive transcriptome data were produced to illustrate the genetics underpinning *C. rotundifolia*’s ecological adaption to seasonal aridity. The modern karyotype and smaller genome of *C. rotundifolia* (*n* = 12, 350.69 Mb/1C), which lack further whole-genome duplication, were mainly derived from gross chromosomal rearrangements such as fusions and segmental duplications, and were sculpted by a very recent burst of retrotransposon activity. Bias in local gene amplification contributed to its remarkable functional divergence from grape, and the specific proliferated genes associated with abiotic and biotic responses (e.g. *HSP-20*, *NBS-LRR*) enabled *C. rotundifolia* to survive in a hostile environment. Reorganization of existing enzymes of CAM characterized as diurnal expression patterns of relevant genes further confer the ability to thrive in dry savannas.

## Introduction

**Figure 1:**
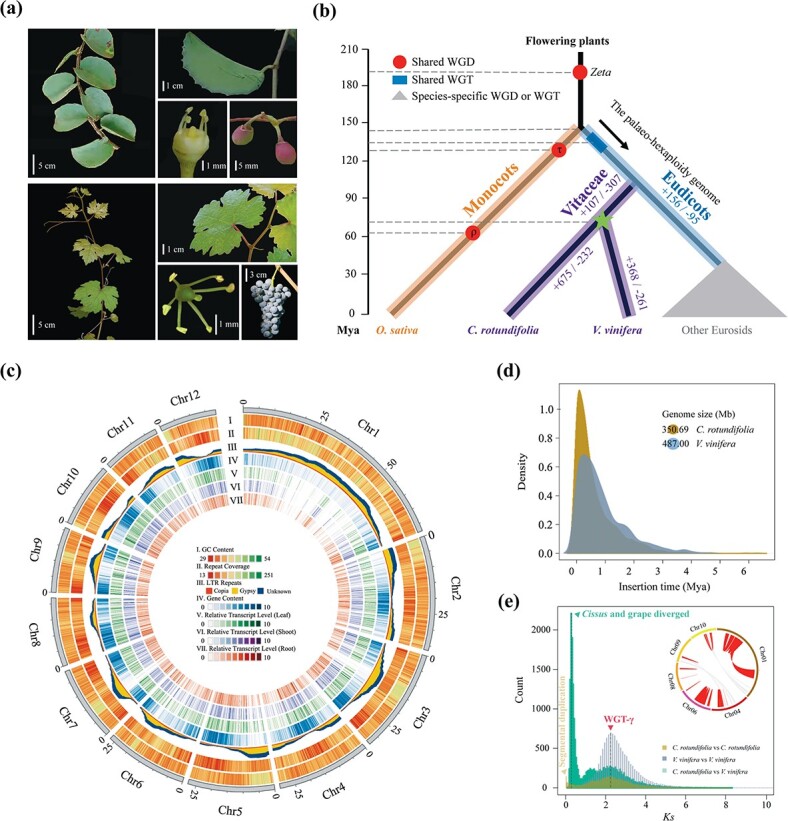
Morphological features and genome evolution of *C. rotundifolia*. **a** The succulent leaf, leaf abaxial surface, flower (without petals), and fruit of *C. rotundifolia* (top). Correspondingly, the vegetative and reproductive organs of grape (bottom). **b** Divergency history between *Cissus* and grape within the phylogeny of flowering plants. Age estimates of each node are based on 342 single-copy genes from 13 representative plant species. WGD or WGT is indicated on the corresponding branches. The numbers of gene family expansions and contractions are indicated along the related branches. **c** Distribution of genomic features of the *Cissus* genome. Each track shows the GC content, repetitive sequence distribution, gene density, and gene expression profile in different tissues from outside to inside. **d** Estimation of LTR activity shows a very recent burst event in *Cissus* <90.77 kya and a much more severe invasion of LTR than in grape. **e** Distribution of *K*_s_ for the whole paranome of *Cissus* and cross-comparison between *Cissus* and grape. Right corner of the image shows the segmental duplications within the chromosomes.

The plant family Vitaceae is well known for its economically important fruit crop, the grape (*Vitis vinifera*). It comprises 16 genera with >950 species and is classified into five tribes [1]. Many species in the family are dominant climbers in tropical/temperate forests, savannas, and mountains [2], representing one of the earliest diverged lineages in the major rosid clade of eudicot plants (Fig. 1a and b) [3, 4]. The grapevine (PN40024) was the first fruit crop species whose genome was decoded [5].

*Cissus* L*.* is the largest genus in Vitaceae, comprising >300 species [1], and the only genus of the tribe Cisseae Rchb. Unlike grapevines, which are mostly distributed in temperate regions, *Cissus* mainly occurs in the seasonal arid regions of the tropics and subtropics [6]. Species in this genus exhibit considerable variation in both chromosome number (2*n* = 24–66) and genome size (1C = 0.38–1.03 pg) [7]. Morphological modifications such as succulent leaves or stems have arisen in some *Cissus* species inthe face of drought stress (Fig. 1a) [8]. Therefore, these groups provide an opportunity to investigate the strategies of plant adaptive evolution with respect to drought tolerance. Crassulacean acid metabolism (CAM) is a water-use-efficient adaptation of photosynthesis that has evolved independently many times in diverse lineages of flowering plants [9]. The genomes of CAM plants, including pineapple (*Ananas comosus*), orchid (*Phalaenopsis equestris*), *Kalanchoe fedtschenkoi*, *Dendrobium catenatum*, *Dendrobium officinale*, and *Sedum album*, are available [3, 10–14]. Comparative analyses between *K. fedtschenkoi* and non-CAM species identified convergence in protein sequence in nocturnal CO_2_ fixation and carbohydrate metabolism [14]. CAM is also widespread in *Cissus* [6, 15–17], enabling us to dissect the convergent evolution of CAM in the plant kingdom.

*Cissus rotundifolia* Lam. is mainly distributed in the tropical savannas of Eastern Africa. The leaves are consumed as a local traditional food [18]. It has a relatively small genome with 1C = 0.38 pg. [7] To understand the adaptive strategies of the genus *Cissus* in the harsh climate, we generated and compared the draft genome of *C. rotundifolia* with that of *V. vinifera* to uncover how the genome has evolved and to identify the genes underpinning its arid adaptation. Further, we conducted extensive transcriptome comparisons to characterize the evolution of CAM in *C. rotundifolia*.

## Results

### Genome assembly, annotation, and repetitive sequence characterization

We assembled a highly heterozygous (1.19%) genome of *C. rotundifolia*, by combining the 39.38 Gb of PacBio Sequel sequences (~106×) and 28.31 Gb of Illumina paired-end reads (~77×) (Supplementary Data Fig. S1, Supplementary Data Table S5). We arranged 3289 contigs (contig N50 = 186 kb) based on the spatial relationship deduced from 130.44 Gb of Hi-C assay data (~362×) (Supplementary Data Table S6). A total length of 350.69 Mb scaffolds was ordered and anchored onto 12 pseudo-chromosomes with scaffold N50 up to 27.6 Mb, covering 94.53% of the assembled genome (Fig. 1c, Supplementary Data Fig. S1, Supplementary Data Table S7). We identified 169 723 homozygous mutation bases representing 0.045% of the assembled genomes (one error per 2.22 kb).

A total of 30 824 protein-coding genes were predicted by using a combination of *ab initio*, transcript evidence, and homology-based methods. We used the Swiss-Prot, NCBI, GO, KEGG, and eggNOG databases to annotate ~82.15% of the coding genes (Supplementary Data Table S8). Moreover, Benchmarking Universal Single-Copy Orthologs analysis suggested that 92.4% of the genes could be recovered (Supplementary Data Table S9). In addition, we identified 692 transfer RNAs, 128 microRNAs, 232 ribosomal RNAs (18S, 28S, 5.8S, and 5S), and 971 small nucleolar RNAs (Supplementary Data Fig. S2).

Repetitive sequences dominated 47.41% of the genome, of which 31.07% were LTR elements (Supplementary Data Table S10). Estimates of sequence divergence times between the adjacent 5′ and 3′ LTRs of the same retrotransposon suggested a very recent burst of activity <90.77 thousand years ago (kya) and much severe invasion than in grape (Fig. 1d, Supplementary Data Table S10). Further, we found 584 679 (12.90 Mb) SSRs with six as the most abundant unit size, slightly less than that in *V. vinifera* (PN40024, 930 680, 23.05 Mb) (Supplementary Data Table S11).

### Gross chromosomal shuffling reassembled the *C. rotundifolia* genome

We collected a total of 342 single-copy genes (61 639 homologous amino acids) among 13 representative angiosperms to clarify the divergence of *C. rotundifolia* (hereafter *Cissus*) and *V. vinifera* (hereafter grape) (Supplementary Data Fig. S3, Supplementary Data Table S1). Reconstruction of the phylogeny indicated these two species had separated as early as 60.19–84.68 million years ago (Fig. 1b), coinciding with the distribution pattern of synonymous substitutions per synonymous site (*K*_s_) (*K*_s_ = 0.33) (Fig. 1e). The subsequent *K*_s_ analysis of all paralogous genes in the genomes of *Cissus* and grape and syntenic regions support a shared whole-genome triplication, namely WGT-γ, the ‘pivot’ paleo-hexaploidy event that occurred in the most recent common ancestors (MRCAs) of all eudicots (Fig. 1b and e) [5, 52]. No more signatures of WGD were observed in *Cissus* and grape genomes. Nevertheless, in *Cissus*, there is another small peak of duplicated genes with *K*_s_ = ~0.1, and the majority of the paired genes were devoid of interchromosomal regions (Fig. 1e, Supplementary Data Fig. S4). Approximately 236 duplication events occurred inside their chromosomes and were characterized as segmental duplications. Such recent local gene cluster duplication burst finally accounted for 8.31% of *Cissus* functional genome profiles (4.75% in grape; Supplementary Data Table S12).

Interestingly, gene ontology (GO) analysis of the segmental duplication gene clusters in two species revealed similar function enrichments mostly associated with basic biological processes, such as phosphorus metabolic process and cellular protein metabolic process. Meanwhile, specific biochemical pathways (e.g. brassinosteroid homeostasis) and chromosome dynamics (e.g. meiotic chromosome condensation and meiotic sister chromatid cohesion) are only enriched in *Cissus* (Supplementary Data Table S12). The role of the ‘connected’ gene cluster as a module of function during speciation and the retention of duplicated segments with gene dosage relationship preserved would be worthy of further investigation [72].

**Figure 2:**
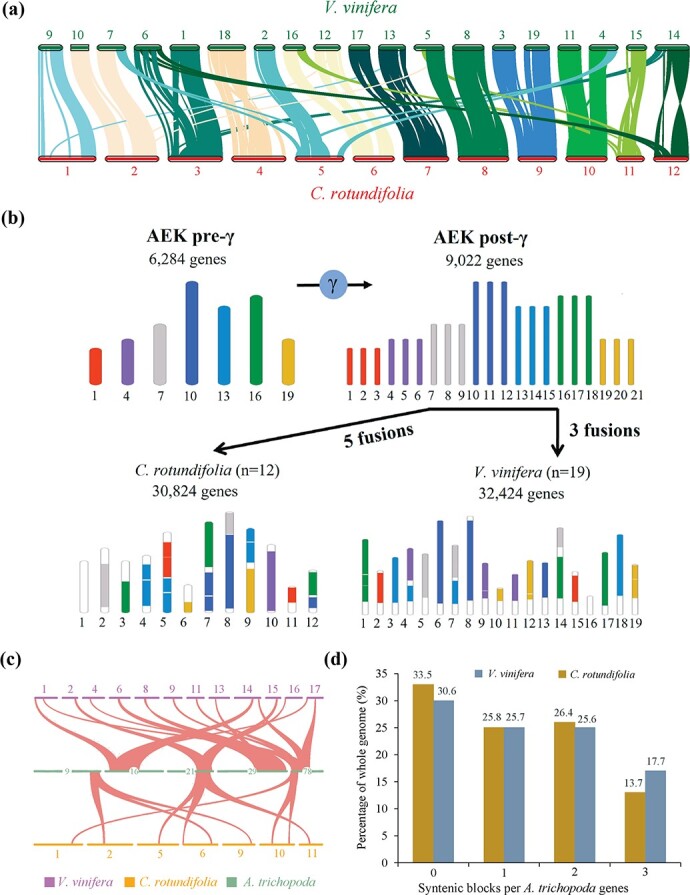
Gross chromosomal rearrangement underlying 12 modern *C. rotundifolia* chromosomes. **a** Macro-synteny patterns between *C. rotundifolia* and *V. vinifera*. The *C. rotundifolia* chromosomes are numbered according to their physical length from long to short. **b** Seven colored codes are used according to the earlier prediction of seven ancient chromosomes of AEK pre-γ, the schematic representation of paralogous regions derived from a grape–cacao–peach comparison (Freeling, 2009) [72]. The karyotypes of *C. rotundifolia* and *V. vinifera* were derived from syntenic comparison with AEK pre-γ and were defined by the occurrence of the syntenic regions as linked clusters in AEK pre-γ, independently of intrachromosomal rearrangements. The evolutionary events were predicted according to the more parsimonious model of evolution. **c** The syntenic relationship among *Cissus*, grape, and *Amborella*. Each *A. trichopoda* scaffold region aligns with up to three regions in either *Cissus* or grape, which are highlighted in red. Shades represent matching gene pairs. **d** Statistics of syntenic regions among *A. trichopoda*, *V. vinifera*, and *C. rotundifolia*. The subset of the proportion of genes in syntenic blocks to the whole genome is indicated on the histograms.

Considerable high collinearity was observed between *Cissus* and grape chromosomes, presenting a pattern of a combination of each two of the 19 chromosomes in grape often corresponding to one chromosome in *Cissus*, leading to fewer monoploid chromosome numbers in the latter (*n* = 12) (Fig. 2a). To search for genomic features that might contribute to *Cissus*’s modern 12 chromosomes, we compared the ancestral eudicot karyotype (AEK) reconstructed from an integration of the Vitales (grape), Malvales (cacao), and Rosales (peach) major subfamilies to uncover that at least five fusions occurred in *Cissus* after inheriting 21 AEK post-γ chromosomes from the MRCA of eudicots (Fig. 2b) [58]. Specifically, pairwise comparisons among *Cissus*, grape, and AEK post-γ revealed that 82.59% of grape genomic regions were linked to AEK post-γ, higher than the number (71.24%) of *Cissus* (Supplementary Data Table S2). This may be partly due to the ancestral reference derived from a comparison of grape–cacao–peach, particularly when the grape preserved more ancestral genomic organizations. Alternatively, the *Cissus* genome may have lost more ancestral gene arrays than the grape, probably attributable to a higher frequency of chromosomal rearrangement and recombination [73, 74]. Beyond that, 17.7% of grape genes (4757 genes) were embedded in syntenic blocks with a 3:1 relationship to each *Amborella trichopoda* [52] region that resulted from WGT-γ, higher than syntenic block genes in *Cissus* genome (13.7%, 3687 genes) (Fig. 2c and d, Supplementary Data Table S3). Likewise, the number of syntenic genes was lower in *Cissus* (19.3%, 4188 genes) than in grape (23.4%, 5089 genes) when aligned with *Aristolochia fimbriata* (a species that is similar to *Amborella* in lacking further WGDs since the origin of extant angiosperms) [75]. Moreover, 76% genes (2419 genes) in chromosome 1 were specific to *Cissus* (Fig. 2b, Supplementary Data Fig. S5, Supplementary Data Table S13). Together with the above, it would imply a more diverged genome of *Cissus* reshaped after long-term separation from *Vitis*.

### Functional divergence enabled *Cissus* adaptation to aridity

**Figure 3:**
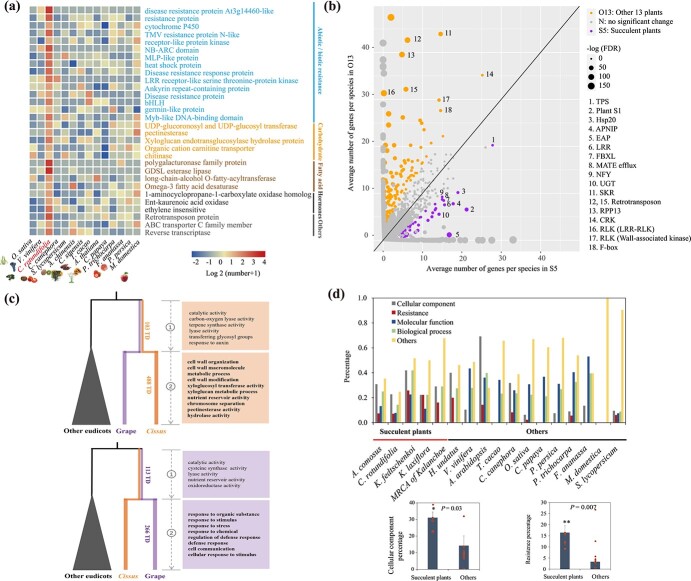
Evolutionary history of functional profiles of *C. rotundifolia* genome. **a** Heat map showing categorized orthogroups that have significantly increased paralogous numbers in *Cissus* compared with other angiosperms analyzed. **b** Scatter plot displaying the expanded orthogroups in 5 succulent plants and 13 other non-succulent plants. Numbers in square brackets associated with circle sizes stand for -log (*P*-adjust), where *P*-adjust is the *P*-value of the binomial test adjusted for multiple testing. 1–18 are terpene synthase, plant self-incompatibility protein S1, hsp20/α crystallin family protein, aspartic proteinase nepenthesin-1 precursor, eukaryotic aspartyl protease family protein, leucine rich repeat protein, F-box domain and LRR-containing protein, MATE efflux family protein, nuclear transcription factor Y subunit, UDP-glycosyltransferase, serine/threonine-protein kinase receptor precursor, retrotransposon protein, disease resistance RPP13-like protein 1, cysteine-rich receptor-like protein kinase, leucine-rich repeat receptor-like protein kinase family protein, retrotransposon protein, wall-associated receptor kinase, and F-box family protein. **c** GO categories with an overrepresented number of tandemly duplicated genes in expanded orthogroups encompassing different evolutionary periods of *Cissus* (upper) and the functional bias of tandem duplicate gene retention in grape (lower). The number of TD events is indicated on the branches. **d** Percentage of GO categories from expanded lineage-specific TD in succulent plants and other non-succulent plants. Cellular component and resistance categories in two subgroups were tested by a two-sample *t*-test.

In *Cissus*, 675 orthogroups were remarkably expanded (*P* < .05) and 232 were diminished (*P* < .05) compared with other representative eudicots (Supplementary Data Fig. S3). The expanded orthogroups were mainly enriched in the abiotic/biotic stress-responsive pathways, metabolism of carbohydrates, and hormone biosynthesis (Fig. 3a). *Cytochrome P450*, found in dramatic proliferation (Supplementary Data Table S14), could contribute to the foliar wax deposition in *Cissus* [76]. Some polysaccharide-related genes, such as pectate lyase, pectinesterase, and polysaccharide biosynthesis genes, also displayed an increased paralogous number, probably attributable to the succulent leaf formation through the modification of pectin and other polysaccharides in cells [8]. Apart from the genes that directly contributed to the leaf character, transcription factors like *MYB*, *WRKY*, *AP2/ERF*, *GRAS*, and *LEA* were strongly expanded (Fig. 3a), suggesting the probability of enhanced abiotic stress resistance and secondary metabolism in *Cissus* [77–79]. To further address the functional divergence of *Cissus* referring to adaptation, we compared its gene repertoire with that of grape, which indicated that selective amplification of genes belonging to plant immunity had occurred in these two species (Supplementary Data Fig. S6, Supplementary Data Table S15). Among respective orthogroups, nucleotide-binding site leucine-rich repeat (*NBS-LRR*) genes were found in favor of expansion in both species but showed preference for different subclasses (e.g. orthogroup 12 in the grape; orthogroup 4,7 in *Cissus*). The co-abundance of *R* genes would represent the basic objective of an organism to protect itself against surging threats from microbial pathogens [80, 81]. The significant copy number variation of paralogous genes (orthogroup 13, terpenoid cyclase; orthogroup 2, TMV resistance protein N-like) likely suggested the different responses to pathogen induction [82, 83]. Additionally, heat shock protein like 20 (HSP20-like) were found particularly amplified in *Cissus* and upregulated in its shoot and leaves compared with root (Supplementary Data Fig. S6, Supplementary Data Table S16). This would fairly reflect the increased ability of *Cissus*’s vegetative organs to deal with heat shock and promote resistance to environmental stress factors [84, 85]. The enrichment pattern of the gene family in *Cissus* led us to investigate if a similar preference for gene proliferation occurred in other succulent species. To this end, we took another four typical succulent plants (*A. comosus*, *H. undatus*, *K. fedtschenkoi*, and *K. laxiflora*) into account in the gene family comparison. We found that 88 of the 97 335 orthogroups demonstrated succulent-specific expansion, which were significantly (*P*-adjust <.05) enriched in ‘*terpene synthase*’ and ‘*HSP20*’ (Fig. 3b, Supplementary Data Tables S17–S19). However, 178 orthogroups that were GO-termed mainly as serine/threonine-protein kinase receptor precursor (*SKR*), cysteine-rich receptor-like protein kinase (*CRK*), and wall-associated receptor kinase (*RLK*) were observed in co-expansion in the other 13 non-succulent plant genomes investigated (Fig. 3b, Supplementary Data Tables S17, S18, and S20). The diverged preference for functional gene families would reflect a specialized convergent mechanism in succulent plants dealing with high temperatures and water deficiency [8]. On the other hand, we identified 1878 tandemly duplicate (TD) arrays of two or more genes in *Cissus*, and the total number of genes in such arrays is 4746, slightly higher than the 3958 genes in 1524 TD arrays in grape (Supplementary Data Table S21). There are 2582 TD genes shared in two species, whose functional classification is mainly enriched in 134 GO terms (e.g. oxidoreductase activity, oxidation–reduction process, and response to auxin), and a total of 2164 TD genes are species-specific in *Cissus* (Supplementary Data Table S22). Functional bias in TD retention was observed encompassing different periods of evolution in *Cissus* (Fig. 3c). An overrepresented number of genes in the *Cissus* lineage were enriched in cell wall-related pathways (e.g. cell wall modification, cell wall organization, and xyloglucan metabolic activity), probably conferring its succulent leaves or stems [86]. In contrast, functional categories specific to grapes were mainly associated with stress responses (Fig. 3c, Supplementary Data Table S23). The result is consistent with the notion that TD genes would have a lineage-specific selection [72]. Nevertheless, earlier studies in *Arabidopsis* and rice demonstrated that an elevated probability of retention of stress-responsive TD is preferential for adaptive evolution after speciation [87, 88]. The functional bias of TD in *Cissus* indicates that genes referred to as morphological innovation for adaptation might be particularly selected and expanded via local duplication. It would be interesting to check if a similar profile of lineage-specific TD is exhibited in other morphology-specialized plants. We found that lineage-TD genes categorized as ‘cellular component-related’ and ‘resistance’ were overrepresented in gross tandem duplicated genes in succulent species, in contrast to the discrete pattern that occurred in the other non-succulent plants (*P* = .03 and *P* = .007) (Fig. 3d, Supplementary Data Table S24). Local gene amplification with a high frequency of gene birth/death plays a critical role in plants’ adaptive responses to environmental stimuli and is mostly attributable to gene copy number and allelic variation within a population [79, 87]. The fashion of TD expansion in succulent plants observed here would suggest another pattern of functional bias in TD retention during seed plant evolution. We speculated that the intense environment change provided multiple options for plants on morphological innovation and rapid expansion of resistance genes.

### CAM photosynthesis in *C. rotundifolia*

**Figure 4:**
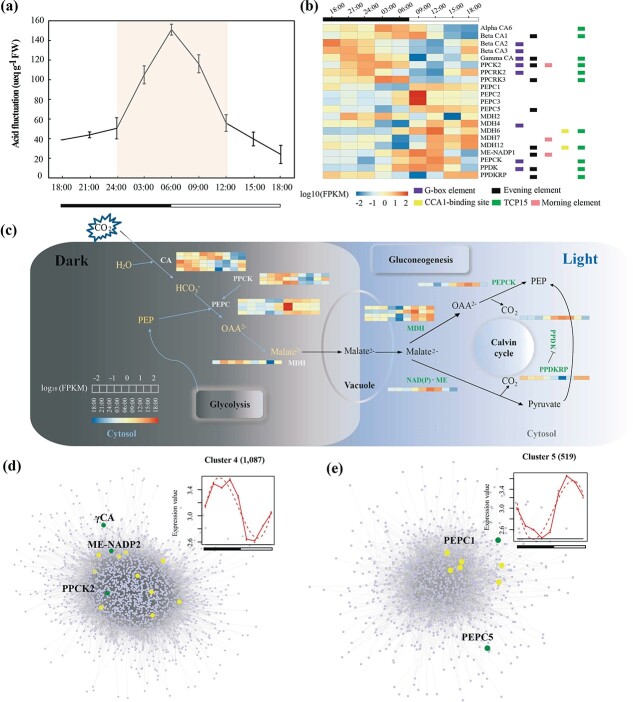
The CAM pathway in *C. rotundifolia*. **a** Diurnal variation of titratable acidity in *C. rotundifolia* leaves*.***b** Expression patterns and *cis*-regulatory elements of CAM-related genes across the diurnal variation. The expression level of each gene is shown using the log_10_-transformed method. The numbers of five circadian clock-related motifs, including G-box element, evening element (EE), morning element (MOM), CIRCADIAN CLOCK ASSOCIATED 1 (ACC1) binding site, and TCP15, are shown in the 2-kb promoter region of each gene. **c** Overview of the CAM pathway. The carboxylation process (dark period) is shown on the left and the decarboxylation process (light period) on the right. Enzymes are marked in blue and green, respectively, with corresponding expression profiles. A network was constructed for Cluster 4 (**d**) and Cluster5 (**e**) using ARACNE. The top 1% of each network is highlighted with a yellow circle, and blue nodes with more than 10 edges were CAM-related genes in *C. rotundifolia*. The yellow and blue nodes are annotated in Supplementary Data Table S29.

CAM photosynthesis is a recurrently evolved strategy for high water use efficiency (WUE), enabling plants to survive in water-limited environments [89]. In CAM plants, carbon dioxide (CO_2_) is fixed in the cytosol and stored as malic acid in the vacuole during the night (Fig. 4c). The stomata remained closed during the daytime to decrease water loss by evapotranspiration, and the stored malic acid is decarboxylated to release CO_2_ that could be re-fixed through the Calvin–Benson cycle [90]. Such a feature of CO_2_ uptake was ubiquitous in the *Cissus* lineage, which may have facilitated the spread of the genus from wet into arid tropics [6].

To investigate CAM evolution in *Cissus*, we determined the pattern of diurnal oscillation of titratable acidity in the leaves of *C. rotundifolia* in growth chambers with a climate close to the dry seasons in Kenya (Fig. 4a). The amount of titratable acid reached its maximum (150 μeq g^−1^ FW) early in the dawn (~6:00) and dropped to its minimum (20 μeq g^−1^ FW) later in the day (~18:00), which qualified *C. rotundifolia* as a CAM species [16, 91]. We identified 47 candidate CAM pathway genes based on their orthologs in pineapple (*A. comosus* L. Merr., CAM plant) [11], maize (*Z. mays* L., C4 plant) [65], rice (*O. sativa* L., C3 plant), *K. fedtschenkoi* (CAM plant) [14], and *P. equestris* (CAM plant) [10]. Further, these genes were well categorized into nine gene families that characterized the core network of carboxylation and decarboxylation pathways (Supplementary Data Table S25) [11]. These gene families showed no significant expansions in *C. rotundifolia* compared with other plants, as shown in Supplementary Data Table S26, implying that CAM photosynthesis might evolve through the re-organization of existing enzymes [69].

The diurnal expression patterns of these CAM genes were interrogated by transcriptome comparison of leaves during 3-hour intervals over a 24-hour period. In general, the expression of 21 genes showed typical circadian patterns as defined via a polynomial regression (Fig. 4b). The transcripts of enzymes involved in carbon assimilation, such as *carbonic anhydrase* (*CA*), *phosphoenolpyruvate carboxylase kinase* (*PPCK*), and *malate dehydrogenase* (*MDH*), were highly accumulated at night. As the core genes involved in CO_2_ fixation, four *PEPC* genes of *Cissus* have extremely high expression in the daytime rather than at nighttime (Supplementary Data Fig. S7). Similar expression patterns were also found in other CAM plants, such as *Kaladp0095s0055.1* in *Kalanchoe* and *Sal_001109* in *S. album* [3, 12, 14, 92]. Correspondingly, the enzymes that participate in decarboxylation processes, such as *MDH*, *ME-NADP*, and *phosphoenolpyruvate carboxykinase* (*PEPCK*) were highly expressed during the day (Fig. 4b). Interestingly, as a major protein for carbon fixation, previous studies in pineapple have shown that only the *βCA* subfamily is expressed at nighttime and early morning in green leaf tissues [11]. We observed all five *CA*s, including α (1), β (3), and γ (1) expressed at night in *C. rotundifolia* (Fig. 4b). The expression of *βCA1* in *Cissus* and pineapple (*R*_cr_ac_ > .8) increased during the night, and a peak occurred at 9:00 in the morning, while its orthologs in *Arabidopsis* (*R*_cr_at_ < .5) showed stable and lower expression during the diurnal cycle (Supplementary Data Fig. S8, Supplementary Data Table S4) [68]. Beyond that, members of *MDH* also showed diverged expression patterns as *MDH2* was more active at night while the other four *MDHs* were upregulated during the day, consistent with other CAM plants (Fig. 4b) [11, 14, 93]. This may be associated with their different roles in decarboxylation processes since *MDH* catalyzes the reversible reaction between oxaloacetic acid and malic acid.

We constructed the gene co-expression network based on the transcriptome data from nine mature leaf samples collected every 3 hours over a 24-hour period. Among 27 modules identified, MEbrown2 (2020 genes that were highly expressed during the night) was significantly (*P* < .05) related to the night period (Supplementary Data Fig. S9). We found that *βCA2*, *βCA3*, and *γCA* were also found in this MEbrown2 module. Pathways such as response to organonitrogen compound and root meristem growth in this module were significantly enriched in this module (Supplementary Data Table S27). The MEdarkorange2 module (311 genes that were highly expressed during the day) was found to be significantly associated with the day period. We found *PEPCK*, *PPDK*, *MDH6*, and *ALMT*s in this module. Biological processes such as response to abiotic stimulus were enriched in this module (Supplementary Data Table S27).

Moreover, transcripts in leaf with time-course diel expression patterns were classified into nine clusters (Supplementary Data Fig. S10, Supplementary Data Table S28). The highly connected hub genes identified by network construction for each cluster were associated with CAM genes. For example, Cluster 4 contained *PPCK2* (CRGY0218762) and *γCA* (CRGY0214246) and had patatin-like phospholipase as the hub (Fig. 4d, Supplementary Data Table S29). Heat shock protein*,* which played important roles during stress responses in many plants*,* was the hub in Cluster5 and connected with *PEPC1* and *PEPC5* (Fig. 4e, Supplementary Data Table S29).

The promoters of the diurnally expressed photosynthetic genes were enriched in circadian clock-related *cis* elements (Fig. 4b) [69, 94]. Comparative analysis between *Cissus*, pineapple, rice, maize, and sorghum showed that only *βCA1* with typical circadian patterns in *Cissus* had one evening element (EE) (Supplementary Data Table S30) [11], suggesting its contribution to CO_2_ fixation via a combination EE motif during nighttime [12]. Additional comparison within CAM genes indicated that EE and G-box elements were mainly enriched in the subgroups of highly expressed genes at night (Supplementary Data Table S30).

The higher WUE in CAM plants relied on the appropriate control of stomatal movement during day and night. We identified the stomata open/close-related genes in the *C. rotundifolia* genome based on their homologs in *Arabidopsis* (Supplementary Data Table S31) [69]. A subset of genes that are responsible for the stomata opening or closing were uniquely expressed either at night or during the daytime, which implied the coincidental organization of stomata movement and CAM genes (Supplementary Data Fig. S11). The expression patterns of stomata movement genes were compared with their orthologs in *Arabidopsis* (Supplementary Data Table S4). We identified 86 out of 141 stomata movement genes with diurnal expression patterns in *Cissus* (Supplementary Data Table S31). The diurnal expression of 64 genes showed a low correlation with its orthologs in *Arabidopsis* (Supplementary Data Fig. S11), suggesting their putative roles during stomatal movement in *Cissus*. *OST1* (Stomatal opening factor1), which plays a vital role in abscisic acid (ABA)-triggered stomatal closure [95], was found to be highly expressed at 9 a.m. (Supplementary Data Fig. S11), compared with accumulated transcription of its orthologs at night in *Arabidopsis*. The result was also consistent with diel expression patterns of *OST1* in *Agave americana*, *K. laxiflora,* and *Kalanchoe* [92, 96, 97]. Interestingly, the MOE and G-box motifs were enriched in the promoter of *OST1* in *C. rotundifolia* but not in *V. vinifera* and *Arabidopsis* (Supplementary Data Table S31). These results indicate the contribution of coordinated transcriptional regulation of circadian in CAM pathway in *C. rotundifolia*.

## Discussion

Vitaceae is a sister to most of the rosids in the highly diverse rosid clade of the flowering plants [98]. Grape (*V. vinifera*) was believed to be one of the most slowly evolved species, representing a more conserved ancestral structure of the genome that can be used to unravel the evolution and genome duplication history of other eudicots [58]. Here, we present another genome in Vitaceae, *C. rotundifolia*, to show a probable diverse evolutionary history considering distinct ecological niches. It is exciting to observe the evidence of the paleo-hexaploidy event shared by *Cissus* and grape. Moreover, the lack of any other WGDs suggests *Cissus* also might hold a relatively ancestral state of genome organization after divergence from the common ancestors [75, 99, 100]. This would be reflected by ~13.7% of the total genes in *Cissus* (~17.7% of grape) were located in the syntenic regions which exhibited ratio of 3:1 to the genomic region of *Amborella*. Besides, we propose that more frequent chromosome shuffling, including at least five independent fusion events, might have occurred in *Cissus* after its origin (three fusions in grape). The recent segmental duplications probably further accounted for the increased genetic and biological complexity [84], together with chromosome fusions serving as a prelude to the modern karyotype configuration of Vitaceae.

The very recent burst of activity in LTRs (90.77 kya) detected in *Cissus* could probably be explained by the severe climate transition from arid to wet that occurred in the past 100 kya in eastern Africa, where substantial ecological habitat turnover was recorded (Fig. 1d) [101]. However, such severe invasion of retrotransposons left a smaller genome (350.69 Mb) than grape (475 Mb). Indeed, we found much higher gene density in *Cissus* than in grape, indicating the lower frequency of repeats in intergenic regions where LTR insertion is usually preferred (Supplementary Data Table S32). Moreover, *Cissus* probably had experienced a fair loss of ancient genomic arrays compared with grape. Together, transposable element removal and sequence elimination accompanied by chromosomal rearrangement (e.g. chromosomal fusion and recombination) could contribute to the selected, size-reduced genome of *Cissus* [73, 74]. Nevertheless, a small genome size could be particularly advantageous for plants to enhance water use efficiency through increased stomatal responsiveness of smaller cells [102, 103].

Seasonal drought is one of the biggest challenges for agriculture in East Africa. The evolution of the water storage tissue of plants is the most common adaptive strategy in arid and semi-arid regions [104]. The leaves of *C. rotundifolia* are succulent, which exemplifies a convergent evolution with plants from dry habitats, like agave [105]. We found gene families of enzymes responsible for polysaccharide synthesis, such as pectate lyase and pectinesterase, were remarkably expanded in *Cissus*. Therefore, modified pectin and other polysaccharides in cells may confer the occurrence of succulent leaves [106]. The noticeable proliferation of gene families associated with biotic and abiotic responses (i.e. *P450*, *LEA*, and *LRR*) would play key roles in the objective arms race against pathogens and unfavorable environments [80, 107, 108].

A clear pattern of selective amplification of immunity genes in *Cissus* and *Vitis* was present, indicating a potential functional divergence related to adaptations. Further, succulent-specific expansion in a certain gene family (e.g. *terpene synthase*, *HSP20-like*) suggested a convergent mechanism in such a morphologically modified group. Interestingly, the fashion of TD expansion in succulent plants was also correlated to morphological innovation, which might unveil another functional bias pattern of TD content in the face of rapid and intense environmental change during seed plant evolution.

The innovation of the CAM photosynthetic pathway in *Cissus* further contributes to its adaptation in the dry savannas by enhancing WUE [109]. The decarboxylation in *Cissus* is likely induced in two ways: one is driven by ME and PPDK enzymes, and another is catalyzed by PEPCK enzyme (Fig. 4c, Supplementary Data Fig. S8). For decarboxylase process, ME and PPDK enzymes were used in *K. fedtschenkoi* and PEPCK enzyme was utilized in pineapple [11, 14]. The genes had undergone convergent evolution in *K. fedtschenkoi*, which included PEPC, nucleosome assembly protein 1-like 4 (NAP1L4), transcription factor hy5-like protein (HY5), and chloroplast-localized glucose-6-phosphate isomerase (GPI). However, no amino acids showed convergent evolution patterns for CAM and stoma-related genes in *Cissus* by a similar analysis [14], suggesting that the evolution of the characteristics may be derived from multiple modifications. In *S. album*, the number variation of *cis* elements between C3 and CAM-cycling status showed a phase shift during the daytime [12], while *cis* elements of CAM cycling genes in *Isoetes howellii* are not strongly associated with transcript expression, additionally lacking ME and G-box on promoters of CAM genes [93]. The EE is over-presented in the promoter of evening-phased genes [110]. Compared with other plants [11], *βCA1* with one EE in *Cissus* may contribute to CO_2_ fixation during nighttime (Supplementary Data Table S30). The identification of *cis*-regulatory elements in the promoter of CAM genes in *C. rotundifolia* would help to explain the evolution of CAM from C3 plants and provide valuable information for breeding drought-tolerant crops.

## Materials and methods

### Plant materials

Stem cuttings of *C. rotundifolia* were collected from Endau hill, Kitui County, Kenya (1°17′49″ S, 38°31′59″ E). Voucher specimens (JAJIT-MU0128) were deposited in the Wuhan Botanical Garden herbarium (HIB). Young leaves of *C. rotundifolia* were collected for genome size evaluation and DNA isolation. Root tips were used for chromosome number determination. Mature leaves, stems, and young roots were collected for RNA isolation and tissue-specific transcriptome analysis.

*C. rotundifolia* individuals were grown in a glasshouse under artificial conditions (16 hours light/8 hours dark, 25°C) in 3-l pots. After being well watered, plants were cultivated for 1 week and transferred to an incubator with 12 hours light (from 6:00 to 18:00) at 30°C and 12 hours dark (18:00 to 6:00) at 20°C (30% humidity). After 3 days, leaves were collected from 18:00 on 9 January 2020to 18:00 on 10 January 2020 at 3-hour intervals. We selected nine time points including 18:00, 21:00, 24:00, 3:00, 6:00, 9:00,12:00, 15:00, and 18:00 for the study. Three biological replicates were collected for each sample and immediately frozen in liquidnitrogen. Samples were stored at −80°C for RNA extraction and titratable acid measurement.

### Estimation of genome size

About 28.31 Gb of Illumina reads were used to evaluate the genome size of *C. rotundifolia* by *K*-mer analysis. The *K*-mer frequency distribution was calculated using Jellyfish (v2.2.6) [19] with the *K*-mer length of 19. Genomic heterozygosity was estimated by GenomeScope (v2.0) [20]. Flow cytometry was used to determine the nuclear DNA content using hand-chopped materials as described by Galbraith *et al*. [21], with minor modifications. Woody plant buffer was used instead of Tris–MgCl_2_ buffer to isolate the nuclei [22]. *Raphanus sativus* cv. ‘Saxa’ was used as the reference standard. Young leaves of *C. rotundifolia* and *R. sativus* were collected, and the protocols were as described in detail by Gichuki *et al*. [24].

### Chromosome count

Root tips were treated with a saturated solution of 1-bromonaphthalene for 3 hours at room temperature (25–28°C) to halt cell division. Microscope slides were prepared from the treated root tips using the protocol developed by Kirov *et al*. [23] and Gichuki *et al*. [24]. The prepared microscopic slides were stained with 4′,-6-diamidino-2-phenylindole (DAPI), and images were captured using a fluorescence microscope (Leica DMi8) fitted with a camera (Leica DFC 550).

### Genome sequencing

Genomic DNA of *C. rotundifolia* was extracted using the CTAB-based protocol described by Doyle and Doyle [25], with minor modifications. Briefly, a washing step was included before CTAB extraction to exclude secondary metabolites. The washing buffer contained 50 mM Tris–HCL, 5 mM EDTA-2Na, 0.35 M d-sorbitol, 1% (w/v) polyvinyl pyrrolidone (PVP-K 30), and 1% 2-hydroxy-1-ethanethiol. Illumina libraries with 450-bp insertions were constructed according to the Illumina standard protocol, and the paired-end libraries were sequenced to ~77 × coverage on the Illumina Hiseq platform. Approximate 20 kb SMRTbell libraries were prepared and sequenced on the PacBio Sequel system following standard protocols (Berry Genomics Corporation, Beijing, China).

### Hi-C library construction and sequencing

Young leaves from the same *C. rotundifolia* plant used for genome sequencing were collected for Hi-C analysis. The Hi-C library construction and sequencing for chromosome-level assembly were implemented by Biomarker Technologies Corporation (Beijing, China) using a previously published method [26]. Briefly, shoot tips of *C. rotundifolia* plants were covered with a black box, and the etiolated leaves were fixed with formaldehyde and lysed. The cross-linked DNA was digested overnight using HindIII. Digested fragments were biotinylated and ligated to form chimeric junctions that were enriched, sheared, and processed. Then libraries were produced on the Illumina Hiseq platform. The paired-end Hi-C reads were uniquely mapped onto the contigs using Juicer [27], and the non-duplicate mapped results were used as the input for the 3D-DNA pipeline [28] to construct the genome sequence. Two rounds (−r 2) provided the best results with an assembly of 12 pseudo-chromosomes and N50 of ~28 Mb. Juicebox was used to fine-tune the assembled genome in a graphic and interaction matrix [29].

### Genome assembly

The contig-level assembly of the *C. rotundifolia* genome was processed by combining ~100× PacBio long reads and ~ 76× Illumina short reads. The raw PacBio reads were initially corrected and trimmed by CANU-1.8 with the parameters of genomeSize = 600 m, useGrid = false, maxMemory = 200 g, ovsMemory = 16G, ovbConcurrency = 15, ovsConcurrency = 15 and batOptions = -dg 3 -db 3 -dr 1 -ca 500 -cp 50 [30]. Then, the corrected PacBio reads were assembled by two widely used PacBio assemblers, CANU-1.8 [30] and WTDBG2 [31]. We used N50 and the genome size of each assembler to inspect assembly quality. The Canu assembled results were adopted in the end. Illumina reads were used to further polish the PacBio assembly using the Pilon program with the following parameters: -verbose -mindepth 4 -fix snps, indels -vcf [32]. Then, the redundancy of the assembled sequences was removed to improve the continuity of the assembled contigs using Redundans with the parameters -identity 0.5 -overlap 0.66 and Purge Haplotigs with the parameters -l 15 -m 60 -h 190 [33, 34].

### Evaluation of the genome assembly

The genome assembly quality of *C. rotundifolia* was evaluated using two methods. Firstly, the error rate considering the homozygous mutation was estimated by mapping the 28.31-Gb whole-genome sequence (WGS) reads onto this assembly by BWA software. Compared with the eudicotyledons_odb10 database, the single-copy orthologs in the assembled genome were identified and the completeness of the assembly was evaluated using Benchmarking Universal Single-Copy Orthologs v2 with the -long and default parameter, respectively [35].

### Repeat sequence annotation

Repeat structures were analyzed by a combined strategy of *de novo* prediction and homology-based prediction. A *de novo* repeat library of the *C. rotundifolia* genome was built by RepeatModeler (v1.0.11, http://www.repeatmasker.org/RepeatModeler/) with the -engine ncbi parameter. Using this library, we processed repetitive sequences to annotate, classify, and mark by RepeatMasker (v4.0.7, http://www.repeatmasker.org/). Two built libraries were combined with the Repbase (v20170127, https://www.girinst.org/) [36] and Dfam (v20170127, http://www.dfam.org/) databases with default parameters [37]. Simple sequence repeats (SSRs) were identified using MISA (http://pgrc.ipk-gatersleben.de/misa/misa.html) [38], with unit length ranging from 1 to 7 and min-length set to 10 bp. Long terminal repeats (LTRs) were identified by LTR_retriever according to the method of Ou and Jiang [39].

### Gene model prediction

Three approaches were combined to annotate protein-coding genes. Firstly, Illumina RNA-seq data from three representative tissues were assembled with two different strategies (*de novo* or genome-guided assembly) by Trinity (v2.2.0) [40]. The assembled RNA-seq data were then aligned to the assembled genome and its evidence-based prediction was performed using PASA (v2.0.2) [41]. Secondly, the *ab initio* methods AUGUSTUS (v3.3) [42], SNAP [43], and GeneMarkHMM (v 4.32) [44] with default parameters were used to predict gene models with training of the best candidate genes obtained from PASA [41]. Thirdly, protein sequences from closely related species, including *V. vinifera* (PN40024) and other *Cissus* species downloaded from NCBI, were used to annotate protein homologs of *C. rotundifolia* by GenomeThreader (https://genomethreader.org/). Finally, the annotation results generated from evidence-based prediction, *ab initio* prediction, and homologous mapping were combined by EVM (v 1.1.1) [45] to integrate the consensus gene model, and genes were renamed according to their position in the genome sequence with the prefix CRGY (*C. rotundifolia* genome).

### Phylogenomic tree construction and gene family analyses

The protein-coding sequences from *C. rotundifolia* and 13 other representative species were used to identify orthologous groups, including those of *Arabidopsis thaliana*, *Oryza sativa*, *V. vinifera*, *Actinidia chinensis*, *Coffea arabica*, *Solanum lycopersicum*, *Populus trichocarpa*, *Theobroma cacao*, *Carica papaya*, *Citrus sinensis*, *Fragaria ananassa*, *Malus domestica*, and *Prunus persica*. All-vs-all BLASTP [46] with an e-value cutoff of 1e−05 was performed, and orthologous genes were clustered using OrthoMCL [47]. Single-copy genes were extracted from the clustering results and multiple sequence alignments were performed using MUSCLE (v 3.8.31) [48]. After removing low-quality alignment or divergent regions by Gblocks, high-quality aligned protein sequences remained. All aligned sequences were concatenated to one long sequence for each species, and these sequences were used to construct a phylogenetic tree by RAxML (v2.5.1) with the PROTGAMMAJTTF model and bootstrap of 1000 [49]. MCMCtree (4.8a) from the PAML package [50] was adopted to estimate the species divergence time according to TimeTree (http://www.timetree.org). Four divergence times were used in this analysis, including those of *C. arabica* and *S. lycopersicum*, *A. thaliana* and *C. papaya*, *P. persica* and *M. domestica*, and *F. × ananassa* and *P. persica*. The divergence times of *V. vinifera* and *C. rotundifolia* in TimeTree were also referenced in this study. The Markov chain Monte Carlo (MCMC) process analysis was set for 50 000 generations and 50 000 burn-in iterations. The OrthMCL results and time divergence tree were used as the input for the CAFÉ (v 3.1) program [51], which was used to identify expansions and contractions of gene families across 15 plant genomes. Family expansion and contraction were analyzed by Count, and the methods and parameters were according to a study of the *Amborella* genome [52]. Multi-species orthologous clusters with gene numbers >0 in *V. vinifera* and *C. rotundifolia* were considered orthologous groups between these two species. Expanded orthogroups were defined according to *P*-value <.05 and a gene number greater than the average value of multiple species. Dot plot representation of orthologous groups was performed with the R package ggplot2 (https://ggplot2.tidyverse.org/) [53].

### Tandem duplication analysis

Four additional typical succulent species, including *A. comosus*, *Hylocereus undatus*, *K. fedtschenkoi*, and *Kalanchoe laxiflora*, were added to the gene family clustering (Supplementary Data Table S1). Multi-species orthologous clusters with a gene number >0 in *V. vinifera* and *C. rotundifolia* were used to identify lineage-specific expansion. Expanded orthogroups were identified with a *P*-value <.05 and gene number greater than the average value of multiple species. The tandem genes were identified by MCScanX [54], which was consistent with the method described in our whole-genome duplication (WGD) analysis. The gained tandemly duplicate (TD) genes of 18 species were obtained from the Count results and were further categorized into either co-expanded or lineage-specific expanded ones. The TD genes were gene ontology (GO)-termed by agriGO database (http://systemsbiology.cau.edu.cn/agriGOv2/index.php). Further, four succulent plants were annotated both by the GO database and the agriGO database.

### Synteny analyses

All-vs-all BLASTP [46] (e-value 1e−05) and MCScanX [54] were used to predict the collinear relationships and positional features between *C. rotundifolia* and *V. vinifera* (PN40024)*.* Blocks of >10 genes and gaps of <5 genes were obtained. The synteny map and dot plot were processed by MCScan and drawn by the python scripts in MCScan packages [55].

The segment duplication events were predicted using self-vs-self BLASTP [46] (e-value 1e−05) and MCScanX [54] among the *C. rotundifolia* genome, requiring at least five genes per collinear block. Subsequently, the pairwise sequences from the synteny blocks and segment duplication pairs were processed by ParaAT (v2.0) [56]. Values of the non-synonymous mutation rate (*K*_a_) and synonymous mutation rate (*K*_s_) were calculated using the NG estimation method in Kaks_Calculator (v2.0) [57]. The visualization plots of the *K*_s_ distribution were made using a custom R script. Additionally, WGD events were determined by the distribution of *K*_s_ of segment duplication pairs and identified by comparisons with the events of *V. vinifera* (PN40024). The ancestral eudicot karyotype (AEK) was inferred from the genomes of eudicot species with the smallest numbers of historical polyploidization events, including grape, cacao, and peach. Further, the AEK was refined as a post-τ ancestral monocot karyotype (AMK) with 10 protochromosomes and 13 916 ordered protogenes, a pre-τ AMK with five protochromosomes and 6707 ordered protogenes [58]. In the current study, the reconstructions of the karyotypes of *V. vinifera* and *C. rotundifolia* were advised by a previous study by Murat *et al*. [58], and the genes and gene orders were used to construct the 7 chromosomes and 21 chromosomes of AEK. To cover as many genes as possible, we used version 2.1 of the grape assembly, which anchored 32 424 coding genes (Supplementary Data Table S1). MCScanX [54], in a BLASTP and dot plot-based approach, was used to detect the syntenic blocks between *C. rotundifolia* versus AEK and *V. vinifera* versus AEK with default parameters. The protein of the pre-γAEK and post-γAEK was compared with *Cissus* and grape by BLASTP. The syntenic blocks were ordered according to the gene order of *C. rotundifolia* and *V. vinifera.* Some small syntenic blocks and small gaps were abandoned or closed to make the syntenic segments more complete. On the base of dot plot illustrations of the synteny between these two species, the karyotypic structures of the ancestral eudicots were explained by taking into account the lowest number of genomic rearrangements, which may have occurred between the AEK and modern eudicot genomes (Supplementary Data Fig. S5, Supplementary Data Tables S2 and S3).

### Detection of significant expansion and contraction in succulent plants

To investigate the significant expansion or contraction of gene families, we divided 18 species into two categories, including five succulent plants (S5) and 13 non-succulent/other plants (O13). Five succulent plants, including *C. rotundifolia*, *A. comosus*, *K. laxiflora*, *H. undatus*, *K. fedtschenkoi*, and other 13 plants are described in Supplementary Data Table S1. The average number of genes per orthogroup between two categories was available to evaluate the significant events. For S5 plants, a binomial test with a probability of success of *P*(W) = 5/18 was used. The criteria of significant expansion or contraction are as follows: (i) a statistically false discovery rate-adjusted *P*-value <0.05 from the initial set of 97 344 orthogroups; (ii) the minimal contribution of about three for S5 and seven for O13 species; (iii) the ratio of contribution to per orthogroup satisfied with (S5n/5)/(O13n/13) > 1. We found that 88 orthogroups were expanded (corresponding to 5696 genes), and 178 were contracted in succulent plants relative to non-succulent plants.

### Measurement of titratable acidity

The diurnal changes of titratable acid in leaves of *C. rotundifolia* were measured as described by Chen and Black [59]. The samples were collected as mentioned above. A total of 0.5 g of leaves of each sample were cut into pieces, placed in centrifuge tubes, and boiled for 30 minutes after adding 10 ml CO_2_-free distilled water. The supernatant after centrifugation was retained. An additional 10 ml CO_2_-free water was added to the pellet to and extraction and centrifugation were repeated. Total supernatants obtained by the two-stage extraction process were titrated to pH 8.3 with 0.01 mol/l NaOH, and the acidity of the leaf was represented in microequivalents of acid per gram fresh weight (μeq g^−1^ FW).

### RNA extraction and RNA-seq library preparation

Total RNA was extracted from the samples using the Universal Plant Total RNA Fast Extraction Kit (BioTeke Corporation, Beijing, China). RNase-free DNase I was used to remove DNA from the extracted RNA. The purity and concentration of RNA were determined by a Nanodrop and Agilent 2100 bioanalyzer (Thermo Fisher Scientific, MA, USA). Subsequently, mRNA enriched by oligo(dT)-attached magnetic beads was randomly fragmented into short pieces with an additional fragmentation buffer. Then, first-strand cDNA was synthesized by random hexamer-primed reverse transcription, followed by second-strand cDNA synthesis. A-tailing mix and RNA index adapters were added. The obtained cDNA fragments were amplified by PCR, and then products were purified using Ampure XP Beads. An Agilent Technologies 2100 bioanalyzer was used for quality control of products. Finally, the cDNA library was constructed, and the MGISEQ-2000 platform was used for paired-end sequencing (2 × 150 bp). Approximately 40 000 000 bp were generated for each sample.

### Transcriptome analysis

The quality of paired-end raw transcriptome data was checked by FastQC v 0.11.8 and trimmed using Trimmomatic (v 0.36) [60]. Then the trimmed reads were mapped onto the latest assembled genome of *C. rotundifolia* using TopHat (v 2.1.1) [61]. Using the gene model of *C. rotundifolia*, the expression levels of genes represented by FPKM (fragments per kilobase million) for each sample were calculated by Cufflinks (v 2.2.1) [61] with default parameters. The genes involved in the stomatal movement process and CAM pathway were picked to show their expression patterns using the pheatmap package in R.

### Identification of CAM pathway and stomatal movement process-related genes

The genomes of *A. comosus*, *V. vinifera* (PN40024), *O. sativa*, *Zea mays*, and *Phalaenopsis equestris* were downloaded from the Pineapple Genomics Database [62], the Phytozome database (https://phytozome.jgi.doe.gov/pz/portal.html) [63], the Rice Genome Annotation Project (http://rice.plantbiology.msu.edu/) [64], MaizeGDB (https://maizegdb.org/) [65], and NCBI (https://www.ncbi.nlm.nih.gov/), respectively. Further, the CAM gene list of *Kalanchoe* was obtained from a supplementary table of its genome [14]. The list of gene families, which included *carbonic anhydrase* (*CA*), *phosphoenolpyruvate carboxylase* (*PEPC*), *phosphoenolpyruvate carboxylase kinase* (*PEPCK*), *malate dehydrogenase* (*MDH*), *malic enzyme* (*ME*), *phosphoenolpyruvate carboxykinase* (*PPCK*), and *pyruvate phosphate dikinase regulatory protein* (*PPDKRP*), was obtained from PGD [62]. All given gene sequences in each family from pineapple, *O. sativa*, and *Z. mays*, *P. equestris* and *Kalanchoe* were used as queries to search corresponding family members in *C. rotundifolia* and *V. vinifera* (PN40024) by BLASTP. The genes with alignment length >100 bp and e-value <1e−05 were considered as potential members. Then online software CD-search (https://www.ncbi.nlm.nih.gov/cdd) [66] and PFAM (https://pfam.xfam.org/) [67] were used to detect the specific domain. The genes without a unique domain of gene family were abandoned. Then the remaining genes were defined as candidate members and used for further analyses. Diel expression datasets of *Arabidopsis* C3 leaf [68] and pineapple CAM leaf were used to compare with CAM genes shown in Fig. 4b in *C. rotundifolia*, whose orthologs were identified by BLASTP based on sequence similarity, and then gene pairs between two species were used to calculate their relationship (Pearson and Spearman) with respect to transcript expression (Supplementary Data Table S4). On the basis of satisfying two correlation coefficients (Pearson and Spearman), genes (*R*_cr-at_ < .5) were determined as not having correlative expression patterns during a day/night cycle between *Cissus* and *Arabidopsis*. Gene pairs (*R*_cr-at_ < .5 and *R*_cr-ac_ > .8) were defined as strongly CAM genes. The genes for stomatal movement were identified using BLASTP with an e-value cutoff of 1e−5 based on orthology in *Arabidopsis* as described by Chen *et al*. [69].

### Co-expression network and cluster analysis

Transcripts with average FPKM >1 (calculated from three biological replicates) in at least one of the nine samples were used to construct a weighted gene co-expression network by the R package WGCNA. The transcript expression was log_2_-transformed. Modules were constructed using the following parameters: power   = 16, networkType = ‘signe’, mergeCutHeight = 0.18, corType = ‘bicor’, minModuleSize = 30. All the nine time-point transcripts with three replicates were used to perform cluster analysis with the maSigPro package. The parameters were as following: degree = 3, counts = F, MT.adjust = ‘BH’. Transcripts were marked as influential by the T.fit() function. Genes with ‘non-flat’ significantly changed across the nine time points. Nine clusters were displayed using the ‘see.genes’ function with cluster.method = ‘hclust’, k = 9 in maSigPro. The network of each cluster was constructed by the ARACNE algorithm with ‘Discovery’ mode and ‘Naïve Bayes’ mutual information (MI) algorithm type in Cytoscape software. The *P*-value was calculated based on MI, in which <.05 was selected in each cluster. One percent of genes with at least 10 edges in each network were selected by cytoHubba, and CAM genes also were chosen based on a minimum of 10 directed edges.

### *Cis*-element annotation and enrichment analysis of CAM-related genes

Promoter sequences in 2 kb upstream of genes involved in CAM were extracted from the *C. rotundifolia* genome. Of all the promoter sequences, the *cis*-element enrichment of light, circadian, temperature, and drought in CAM-related and stomatal movement-related promoters were implemented by the FIMO [70] program with *P*-value <.0002 in MEME. Enrichment analysis of about five known *cis*-elements, including the morning element (CCACAC), the evening element (AAAATATCT), the CCA1-binding site (AAAAATCT), the TCP15 element (NGGNCCCAC), and the G-box element (G-box; CACGTG) [71], was performed by the FIMO program [70].

## Acknowledgements

This work was supported by the National Science Foundation of China (31961143026) and the Scientific Research Program of Sino-Africa Joint Research Center (SAJC201614 and SAJL201607). We would like to thank the Institute of Experimental Botany, Czech Republic, for kindly providing the seeds of *R. sativus* cv. ‘Saxa’ as the standard for flow cytometry.

## Author contributions

H.P.X. and Q.F.W. initiated the study of the *C. rotundifolia* genome sequencing project. H.P.X., Y.W., Q.Y.L., and T.W. are joint first authors. D.K.G. and Z.F.Z. confirmed the genome size and chromosome numbers with help of B.L. D.K.G. and H.M.Z. isolated DNA. Y.W. and Q.Y.L. carried out the genome assembly, annotation, and transcriptome analysis with the help of J.S.Z. Y.W. carried out the phylogenomic analyses with the help of Y.D.Z., B.L., T.W., and Z.D.C. Y.J.H., Y.S.L., and C.X. detected the diel acid fluctuation in the leaves of *C. rotundifolia*. Y.S.L., R.J.L, Z.M.L., and Q.Y.L. identified the CAM pathway-related genes. Y.J.H. isolated the total RNAs, and Q.Y.L. performed the expression pattern analysis of CAM pathway related genes with the help of H.S.J. H.P.X., Y.W., Q.Y.L., J.W. and Q.F.W. wrote the initial manuscript. J.W., J.N.W., Z.D.C., Z.C.L., L.M.L., G.W.H., R.J.L., R.A. and R.W.G. contributed to the discussion of project at different stages. All authors revised and contributed to the final version of the text.

## Data availability

All data used and generated in this study have been deposited in the National Genomics Data Center (NGDC, https://ngdc.cncb.ac.cn/) with the project number PRJCA005006. The final assembled genome and annotation files were also deposited in www.grapeworld.cn/ggh/cis.html. All data is available from the corresponding author upon reasonable request.

## Conflict of interest

The authors declare no competing interests.

## Supplementary data

Supplementary data is available at *Horticulture Research* online.

## Supplementary Material

Web_Material_uhac208
