# Supplementary material for: A genome for Cissus illustrates features underlying its evolutionary success in dry savannas
Source: Hortic Res. 2022 Sep 13;9:uhac208. doi: 10.1093/hr/uhac208 (PMC9715578; doi:10.1093/hr/uhac208)
Supplement: Web_Material_uhac208 [file web_material_uhac208.zip › 3. Supplementary Information-Cissus genome (final).docx]

**Title:**

**A genome for *Cissus* illustrates features underlying the evolutionary success in dry savannas**

Haiping Xin^1,2,3#^, Yi Wang^4#^, Qingyun Li^1,2,3,5#^, Tao Wan^1,3,6#^,Yujun Hou^1,5^, Yuanshuang Liu^1, 5^, Duncan Kiragu Gichuki^1,5^, Huimin Zhou^1,5^, Zhenfei Zhu^1,5^, Chen Xu^1^, Yadong Zhou^1,2,3^, Zhiming Liu^6^, Rongjun Li^1,2,3^, Bing Liu^3,7^, Limin Lu^3,7^, Hongsheng Jiang^1^, Jisen Zhang^8^, Junnan Wan^1,2,3^, Rishi Aryal^9^, Guangwan Hu^1,3^, Zhiduan Chen^3,7^, Robert Wahiti Gituru^10^, Zhenchang Liang^3,4*^, Jun Wen^11*^, Qingfeng Wang^1,2,3*^

**Supplemental Figures**

**
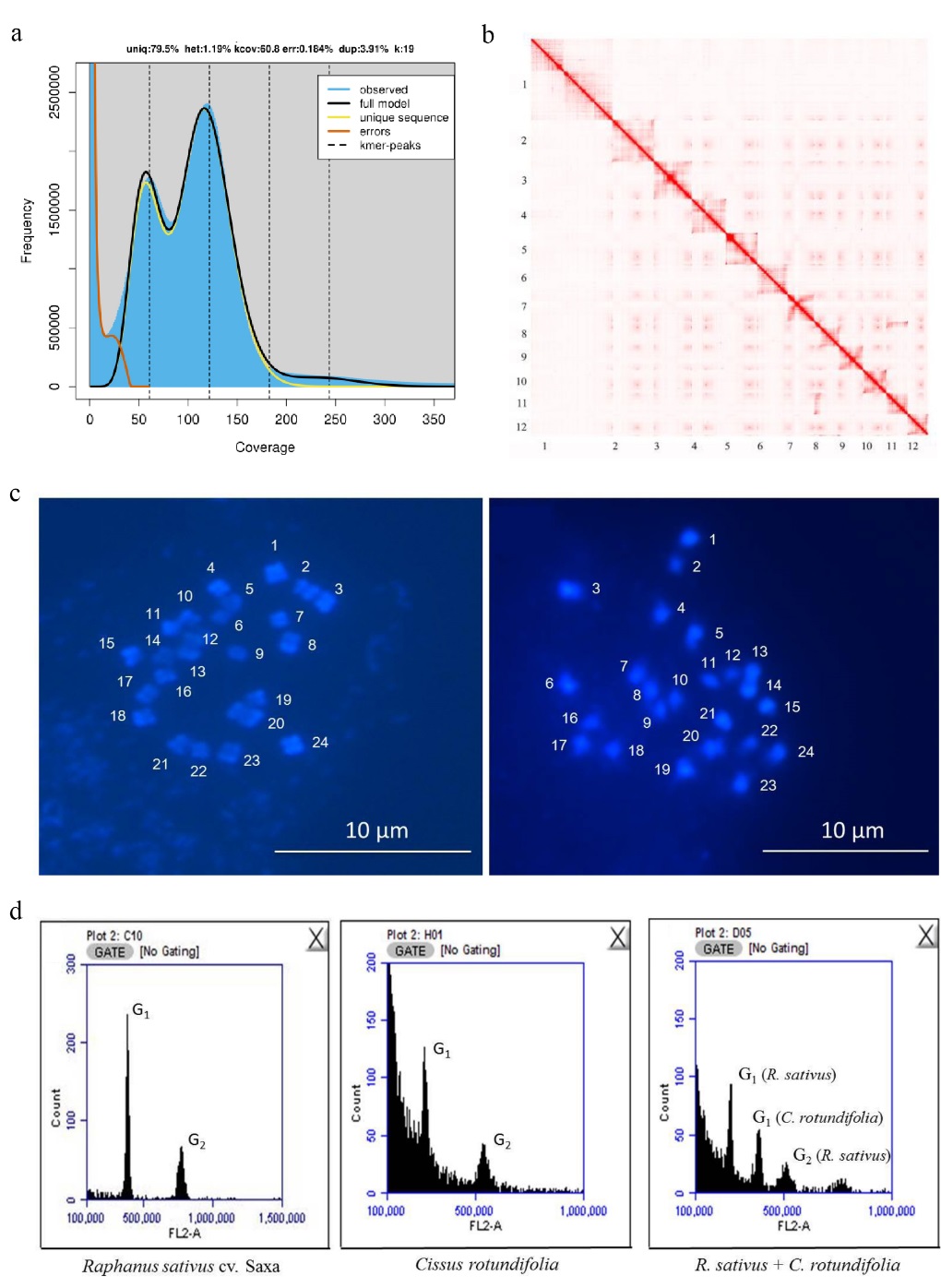
**

**Figure S1 The chromosome number, genome size, heterozygosity and Hi-C contact map of *Cissus rotundifolia.* a,** The genome size and heterozygosity survey according to the *K-mer* abundance distribution (*K-mer* = 19). **b,** The genome-wide chromatin interactions in *C. rotundifolia*. **c,** Mitotic metaphase chromosome complements from root tip cells of *C*. *rotundifolia*. Bar = 10 µm. **d,** Fluorescence histograms for genome size assessments in *C. rotundifolia* by flow cytometry, from left to right, *Raphanus sativus* cv. Saxa (Radish, 2C = 1.1 pg) was used as internal reference standard. The fluorescence peaks from G_1_ and G_2_-phase nuclei were marked as G_1_ and G_2_. *C. rotundifolia* with G_1_ and G_2_ peaks. Pooling samples from *R. sativus* cv. Saxa and *C. rotundifolia*. The relative fluorescence intensities of G_1_ from two species in pooling samples were used to estimate the genome size of *C. rotundifolia* (about 360 Mb which calculated from three replicates).


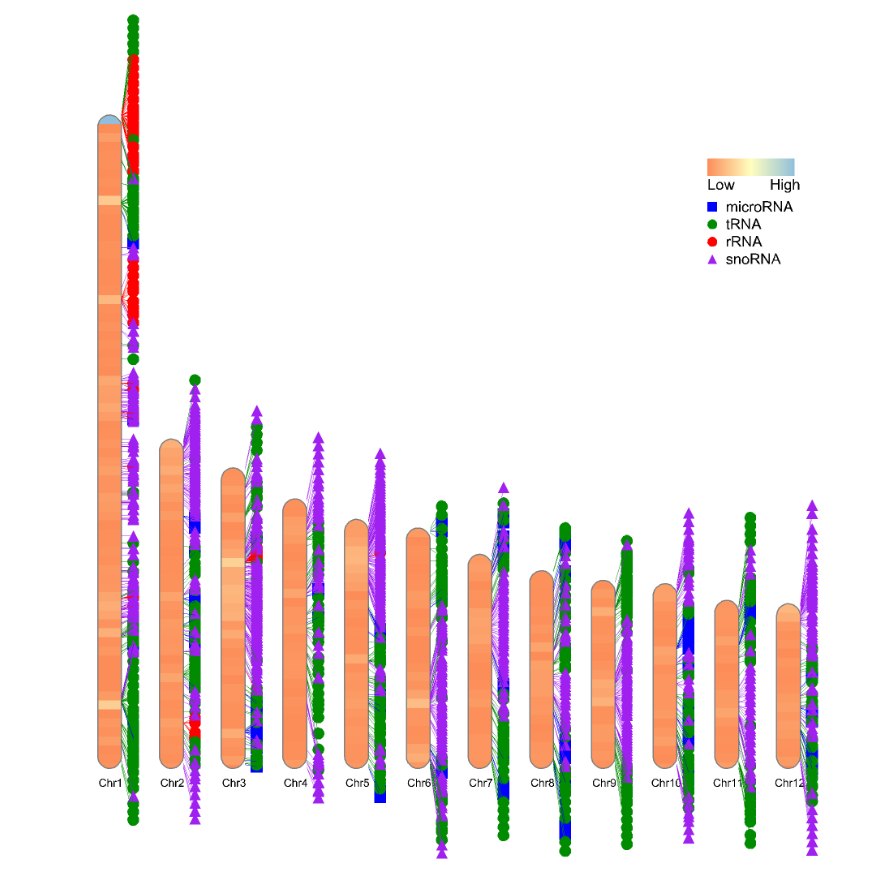


**Figure S2 Distribution of microRNA, tRNA, rRNA and snoRNA in each chromosome of *C. rotundifolia*.**


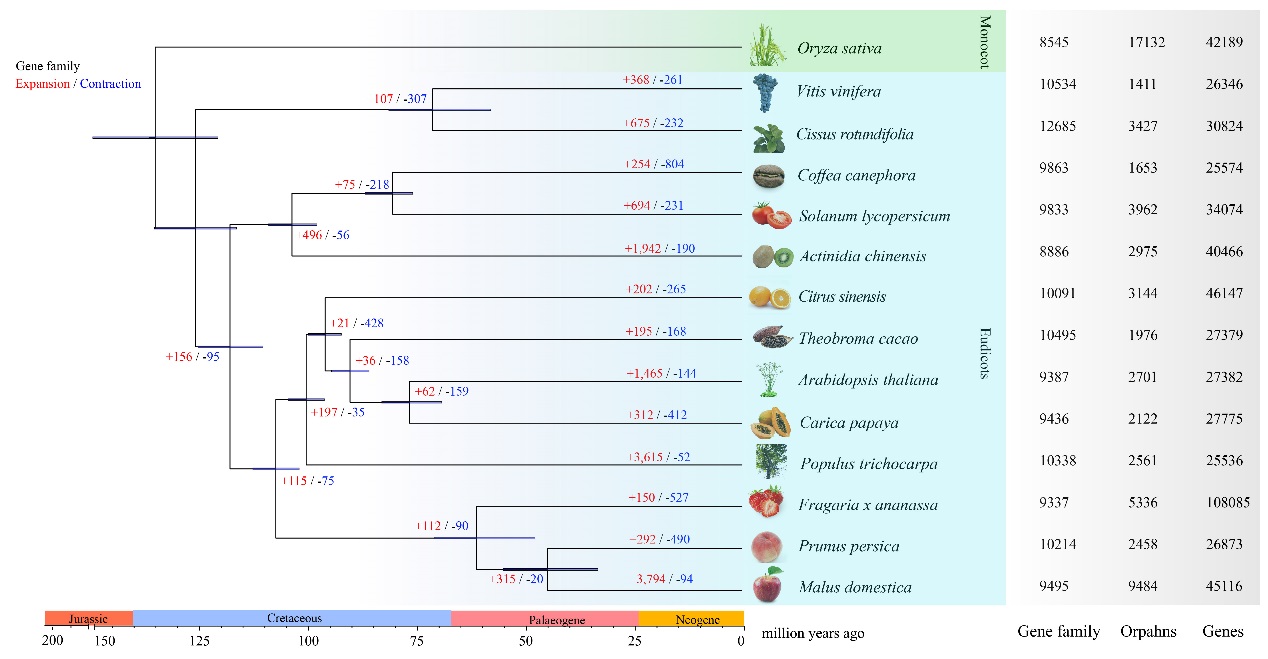


**Figure S3 Phylogenetic tree showing gene family expansion/contraction analysis compared with 13 representatives of the eudicot plants.** The number of gains and losses are indicated along the nodes and branches. The number of gene families, orphan (single-copy gene families) and annotated coding genes is indicated next to each species. Standard errors for estimates of node ages are given for each node.


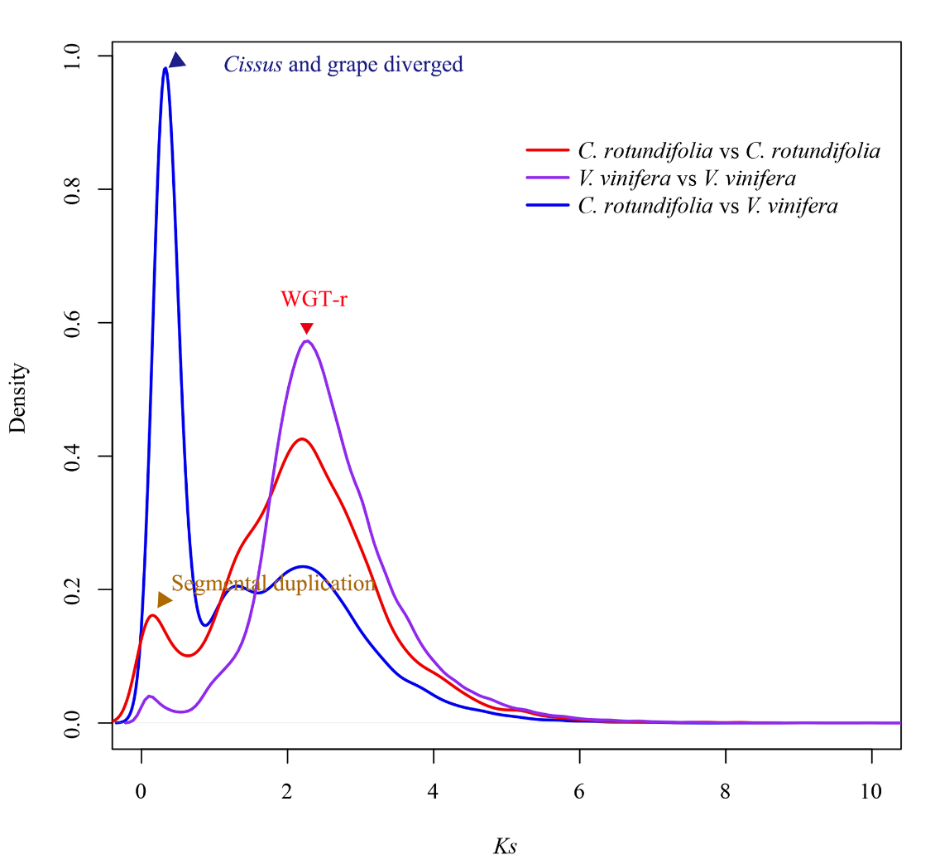


**Figure S4 The density distribution of *Ks* of *V. vinifera* vs *V. vinifera*, *C. rotundifolia* vs *C. rotundifolia* vs and *V. vinifera* vs *C. rotundifolia*.** The three peaks indicted segment duplication, species divergency and WGT-r events in respective.


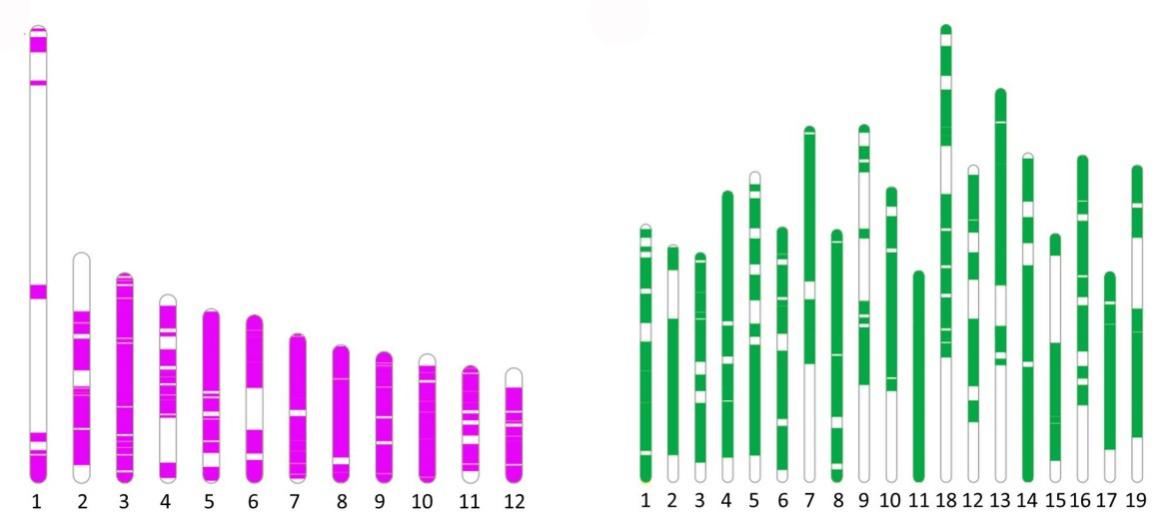


**Figure S5 The highly conserved regions between the genomes of *C. rotundifolia* and *V. vinifera* cv. Pinot Noir (PN40024).** Compared with *V. vinifera*, most of the chromosomes of *C. rotundifolia* composed by the conserved regions between two species, which indicated the loss of the genome fragments during chromosome fusion events in *C. rotundifolia*.


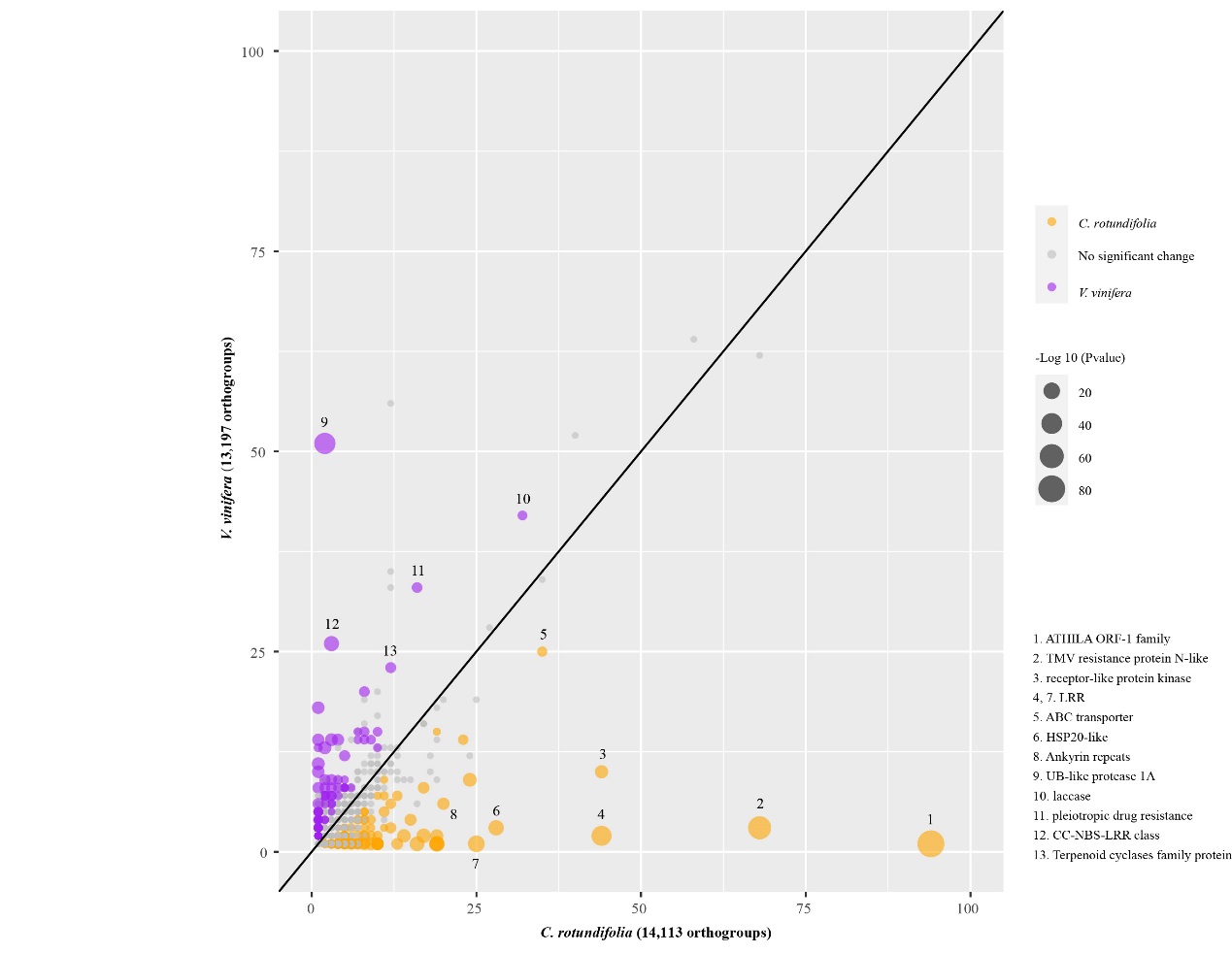


**Figure S6 Scatter plot displayed the expanded orthogroups in *C. rotundifolia* and *V. vinifera*.** Numbers in square brackets associated with circle sizes stand for –log (pvalue).


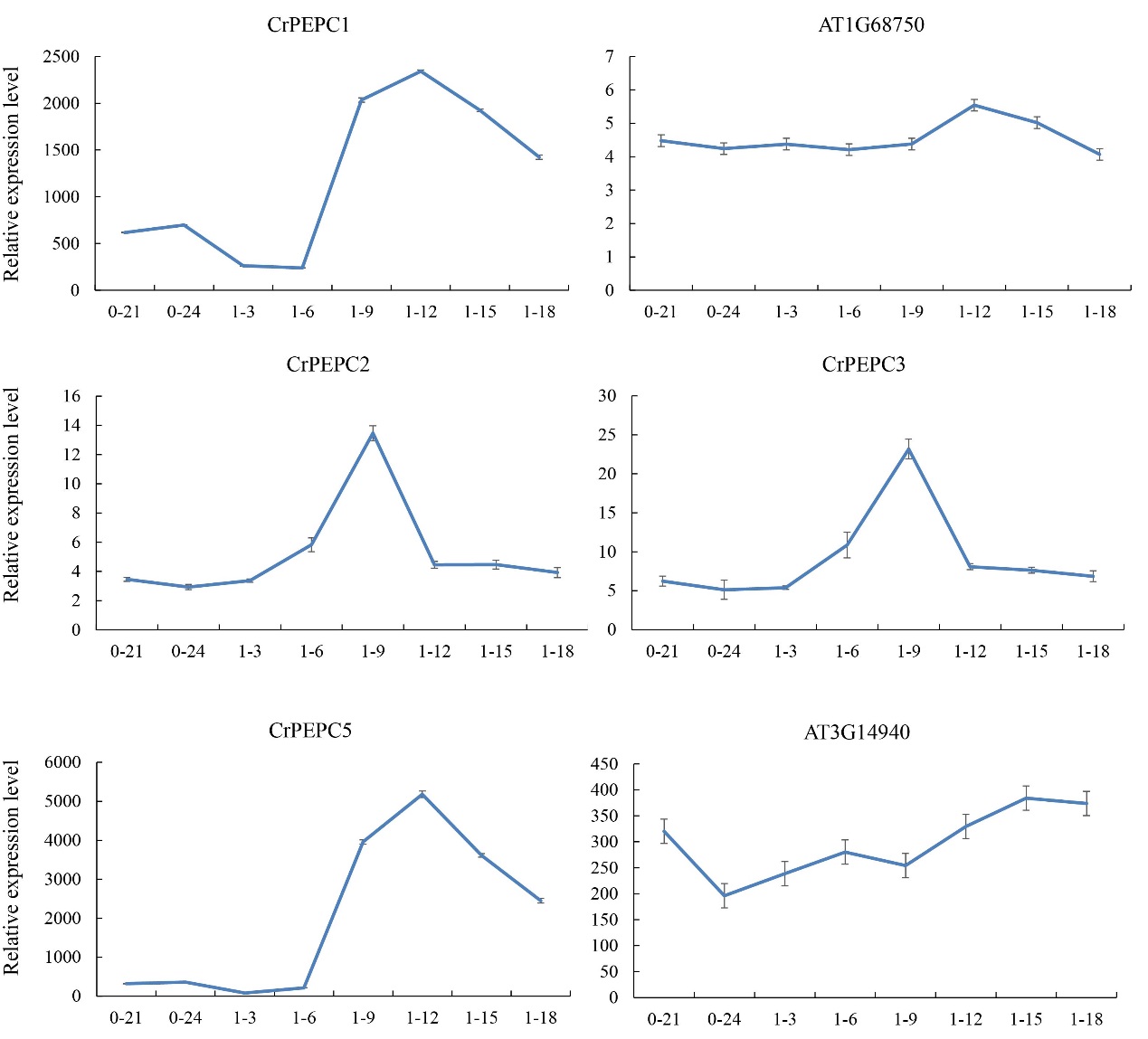


**Figure S7 The relative expression level of four PEPC genes in *C. rotundifolia* and two orthologs in *Arabidopsis*.** The relative expression levels of PEPC1 and PEPC5 orthologs in *Arabidopsis* (AT1G68750 and AT3G14940).

**
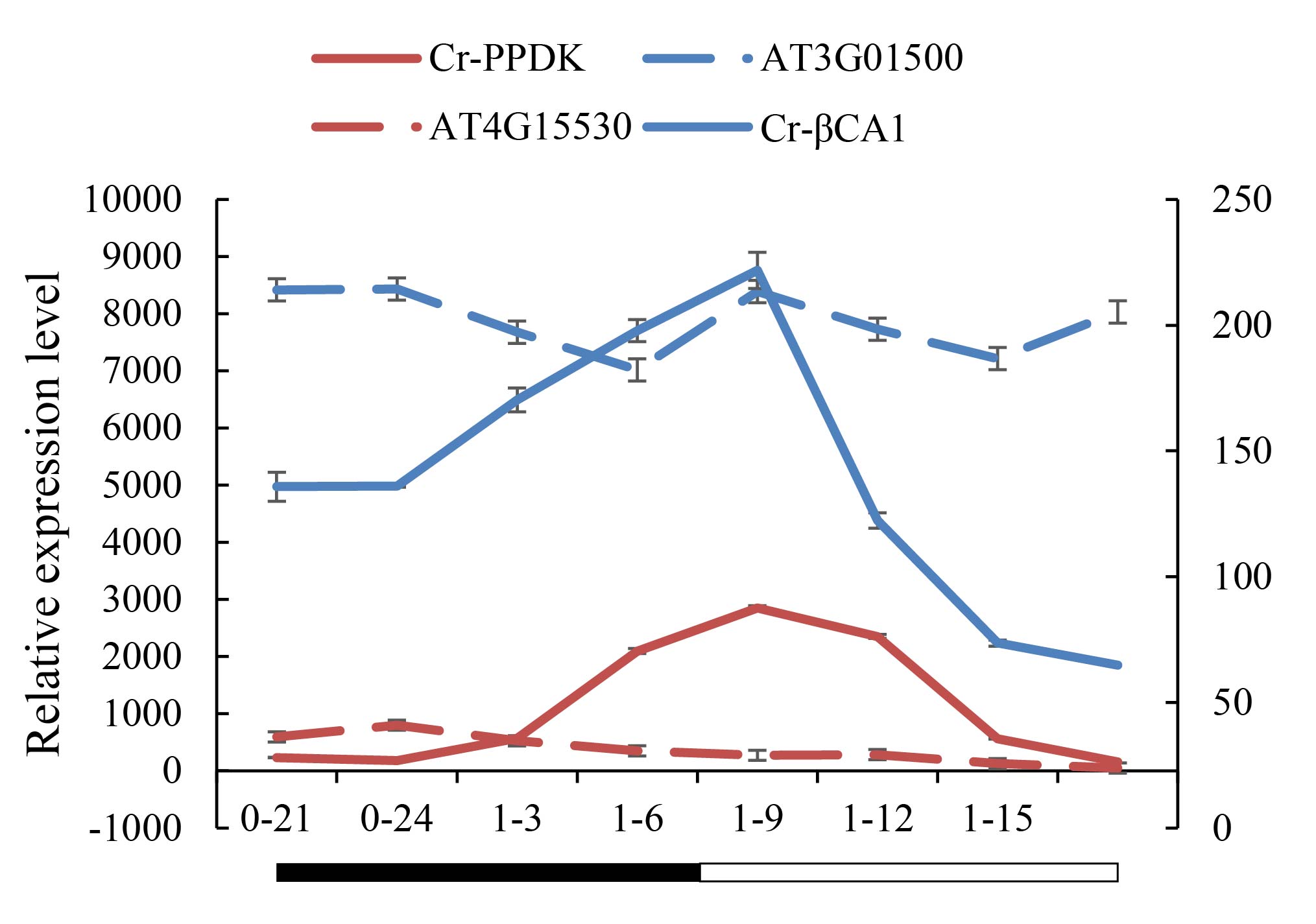
**

**Figure S8 The relative expression level of βCA1 and PPDK in *C. rotundifolia* and two orthologs in *Arabidopsis*.** The abundances of Cr-PPDK and AT3G01500(AtβCA) were showed in left Y-axis and the abundances of βCA1 and AT4G15530 (AtPPDK) were showed in right Y-axis.

**
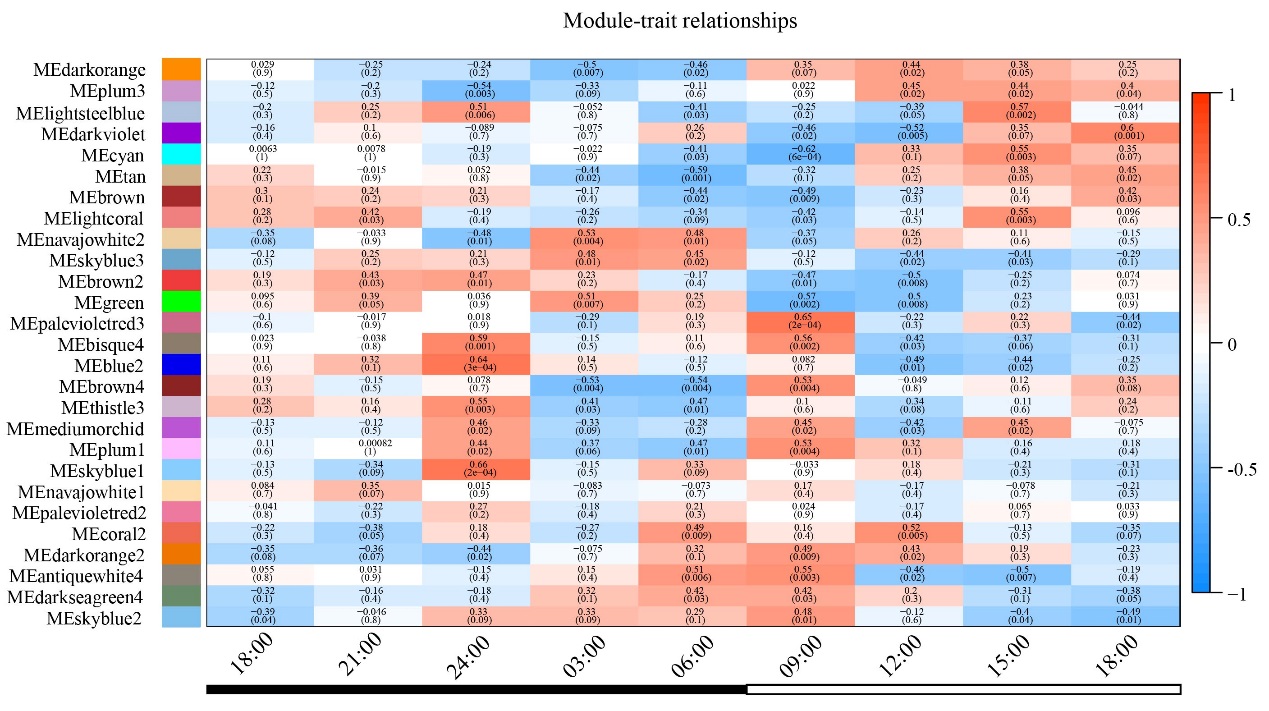
**

**Figure S9 The module and phase relationships of dial expressed genes in *C. rotundifolia*.**

**Figure S10 The clusters of dial expressed genes using gens by** **maSigPro package in *C. rotundifoli*
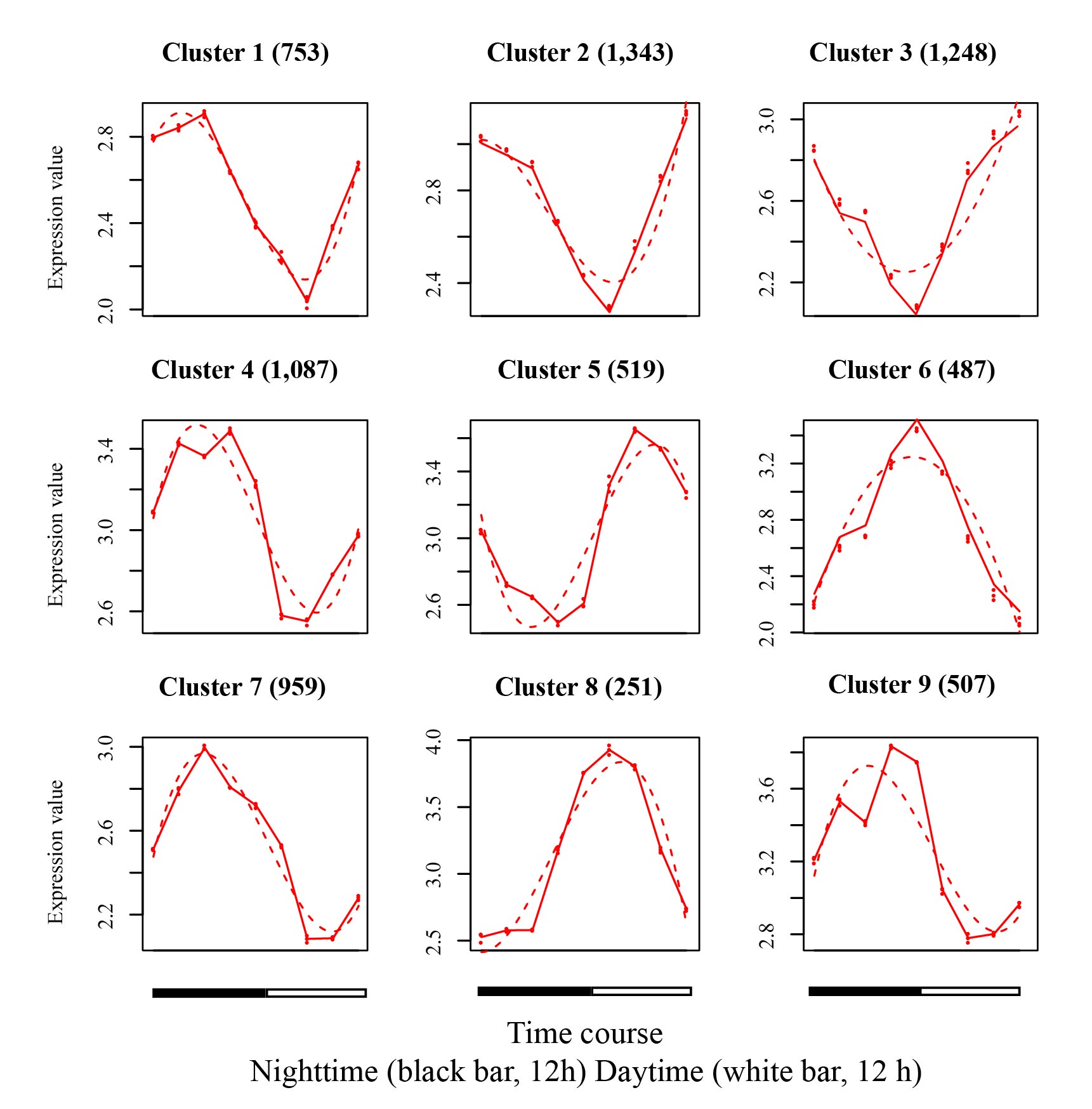
*a.***


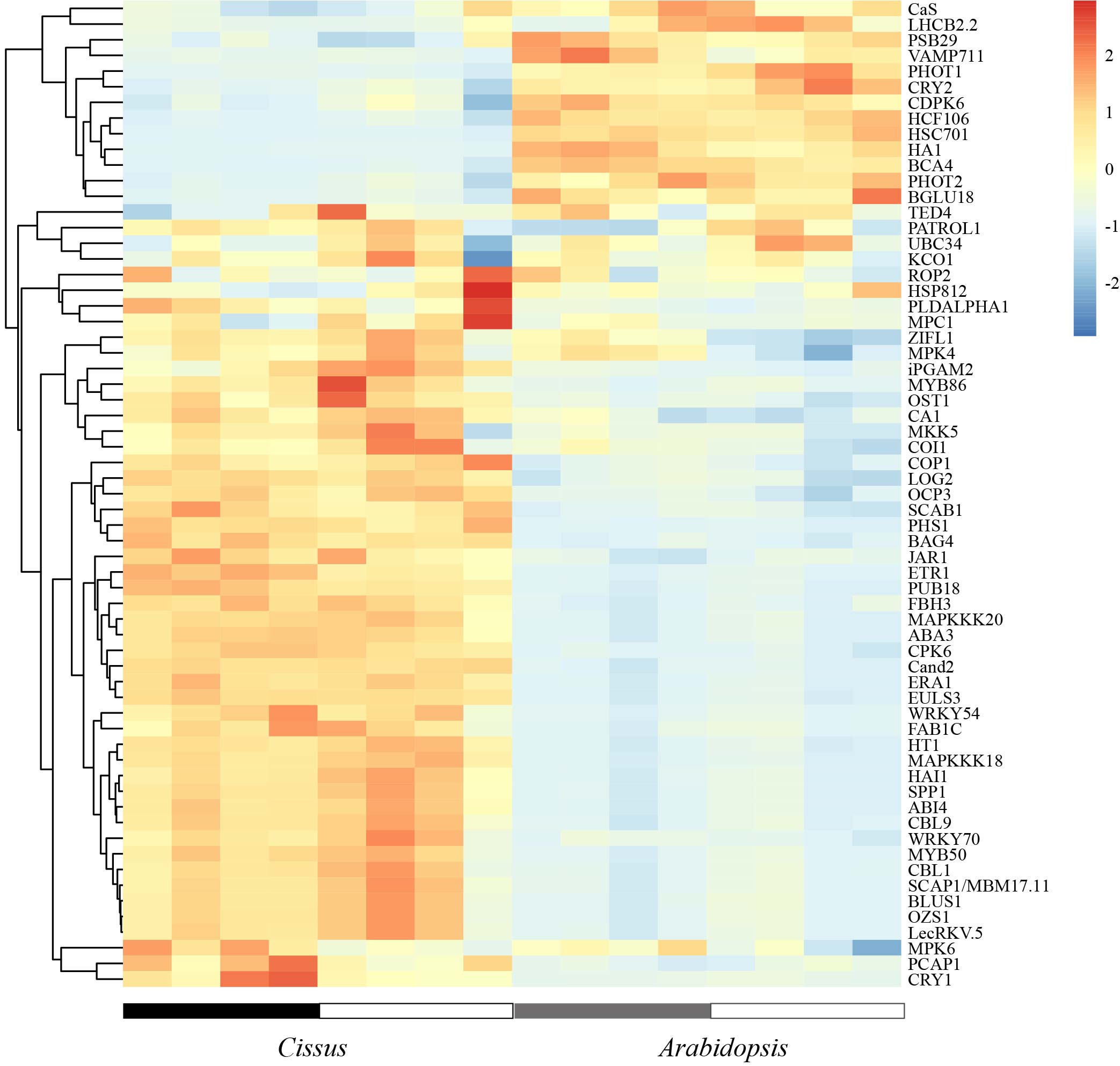


**Figure S11 The dial expression pattern of a subset of genes that be responsible for the stomata open or close in night and day.** Left panel represents stomatal movement genes in *Cissus* and right panel represents orthologs in *Arabidopsis* genes based on marked ‘not-flat’ and correlation coefficient < 0.5 between these two species.
